# Young people’s choice and voice concerning sex and relationships: effects of the multicomponent Get Up Speak Out! Programme in Iganga, Uganda

**DOI:** 10.1186/s12889-022-13919-x

**Published:** 2022-08-23

**Authors:** Tasneem Kakal, Christine Nalwadda, Miranda van Reeuwijk, Maaike van Veen, Lincie Kusters, Ophelia Chatterjee, Charles Owekmeno, Maryse Kok

**Affiliations:** 1 KIT Royal Tropical Institute, Amsterdam, The Netherlands; 2 Makerere University, Kampala, Uganda; 3 Rutgers WPF, Utrecht, The Netherlands; 4 SRHR Alliance, Kampala, Uganda

**Keywords:** Sexual and reproductive health and rights, Adolescents, Youth, Uganda, Voice, Choice

## Abstract

**Background:**

Young people in Uganda face challenges in achieving their sexual and reproductive health and rights (SRHR), such as lack of information, limited access to services, teenage pregnancy and sexually transmitted infections. To address this, their empowerment – including their ability to express themselves and make decisions, is a key strategy. This study assessed how young people’s voice and choice concerning sex and relationships changed over the period of 3 years of implementation of the Get Up Speak Out! programme.

**Methods:**

Data were collected through a household survey with young people (15-24 years) and through focus group discussions, in-depth interviews and key informant interviews with youth and community stakeholders in 2017 for the baseline and 2020 for the end-line. Using the difference-in-difference technique and thematic analysis, changes in key outcomes were assessed over time between intervention and control area.

**Results:**

There were limited changes over time in the intervention area, which did not differ from changes in the control area. Young people were able to express themselves and expand their decision-making space on sex and relationships, in particular if they were older, male and in a relationship. Young women negotiated their agency, often by engaging in transactional sex. However, youth were still restricted in their self-expression and their choices as speaking about sexuality was taboo, particularly with adults. This was influenced by the political and religious climate around SRHR in Uganda, which emphasised abstinence as the best option for young people to prevent SRHR-related problems.

**Conclusions:**

Young people’s SRHR remains a challenge in Uganda in the context of a conservative political and religious environment that reinforces social and gender norms around youth and young women’s sexuality. The limited effect of the programme on increasing young people’s voice and choice concerning relationships in Uganda can be understood in the context of a ban on comprehensive sexuality education (CSE) and the COVID-19 pandemic. These structural and emerging contextual factors enforce the taboo around youth sexuality and hinder their access to SRHR information and services. Multi-component and targeted programmes are needed to influence changes at the structural, community and individual level.

**Supplementary Information:**

The online version contains supplementary material available at 10.1186/s12889-022-13919-x.

## Background

Since the International Conference on Population and Development (ICPD) in 1994, progress has been made in improving sexual and reproductive health and rights (SRHR), including for young people (10-24 years) [1]. At the same time, the agenda of ensuring SRHR of young people as part of the ICPD Programme of Action remains unfinished [2].

Strengthening young people’s empowerment has been widely recognized as an important strategy in attaining their SRHR. Young people’s empowerment can be defined as “the expansion of choice and the strengthening of voice through the transformation of power relations” [3]. Agency is at the centre of empowerment. It is about young people’s “capacity for purposive action, the ability to pursue goals, express voice and influence and make decisions free from violence and retribution” [3]. To expand young people’s voice and choice concerning sex and relationships, a supportive environment is needed: in terms of access to comprehensive SRHR information and services, supporting families and wider communities, and (national level) laws and policies. However, this support is often constrained by conservative norms around young women’s and men’s sexuality and unequal power relations between adults and young people, men and women, and people with different intersecting social backgrounds and contexts [4, 5].

It is noteworthy and urgent to recognize that for young women in particular, empowerment, and the right to exercise their voice and choice, is limited. For example girls and young women, might not be able to make voluntary and informed choices on when or with whom to have sex, start a relationship or marry; and on contraceptive use. As a result, sexually transmitted infections (STIs), teenage pregnancy, unsafe abortion, maternal death and disability, and gender-based violence – including child marriage – are major problems that young people face, particularly young women, in sub-Saharan Africa [6]. Moreover, young people face barriers in being involved in the design, implementation and evaluation of programmes by governments and development partners [7]. Young women’s choices and voice can be further constrained depending on their race, class or other identities [3].

Uganda is the most youthful country in East Africa: over half (55%) of the population is below the age of 18 and 35% of the population comprises 10-24 years olds [8]. Although Uganda’s National Health Policy specifically addresses sexual and reproductive health (SRH) needs of youth and a National Adolescent Health Policy is available, young people’s SRHR need more investment [9]. Despite improved political will to address sexuality education in school, resistance persists from selected conservative religious and cultural leaders [10]. Twenty-five percent (25%) of women aged 15-19 years have begun childbearing [8]. While knowledge of contraceptive methods is nearly universal in Uganda, 28% of currently married women and 32% of sexually active unmarried women have an unmet need for contraception [8]. Sexually transmitted infections, including HIV, are also common. Young women aged 15-24 years are 2.5 times more likely to be affected by HIV (5%) than men of the same age (2%) [11]. Moreover, 50% of ever-partnered women aged 15-49 years have experienced intimate partner physical and/or sexual violence at least once in their lifetime [8]. Most recently, the Ugandan government banned comprehensive sexuality education (CSE) in 2016, and released new guidelines on sexuality education which focused on abstinence [12].

A multitude of factors contribute to these SRHR problems among young people in Uganda. These factors include: early sexual debut; transactional sexual relationships; peer pressure; inadequate information on SRHR from parents, teachers, and health workers; unacceptance of young people’s use of contraceptives; fear of side effects or misconceptions about contraceptives; and cultural and gender norms, such as norms favouring early motherhood and having many children [13–15]. In a survey involving sexually active adolescent girls (15-19 years) in 16 districts of Uganda, the authors found that knowledge about condoms did not result in consistent and correct use of condoms, and the same was found for other contraceptive methods. The authors argued that, as found in other studies, a taboo on youth sexuality, in particular for girls and young women, constrains young people’s (sexual) agency [16]. Adolescent-parent communication on SRHR is limited, and where present, focuses on STIs, bodily changes and abstinence rather than dating and sex [17], despite some adolescents starting sexual relationships at 15 years [8]. The COVID-19 pandemic has also resulted in reduced access to comprehensive sexuality education and information and family planning services in the country [18].

The Sexual Reproductive Health and Rights Alliance (SRHR-Alliance) in Uganda, a consortium of eight organizations^1^ founded in 2011, has implemented the Get Up Speak Out (GUSO) programme in four districts: Iganga, Bugiri, Mayuge and Jinja (2016-2020). The programme aimed to ensure that “all young people, especially girls and young women, are empowered to realise their SRHR in societies that are positive towards young people’s sexuality” [19, 20]. The programme, using a multi-component approach, aimed at improving young people’s ability to make their own decisions and voice their rights, increasing access to SRHR information and SRH services, and creating an enabling socio-cultural, policy and legal environment. More details about programme activities are provided in Table 1. This study aimed to assess how young people’s voice and choice concerning sex and relationships changed over the period of 3 years of the GUSO programme implementation.

**Table 1:** An overview of GUSO programme activities

The GUSO programme used the multicomponent systems approach whereby the provision of SRHR information and education was linked to increasing quality and youth-friendly SRH services and at creating an enabling and supportive environment [19]. Interventions under the GUSO programme included, amongst others, the following: - SRHR and CSE training of peer educators and teachers - Provision of CSE in- and out of school - Online SRHR information provision and campaigns on various platforms and social media - Intergenerational dialogues on SRHR in communities, attended by community leaders, religious leaders, parents and young people - Advocacy on local, district and national level to implement SRHR policies and laws - Training of services providers and peers on youth-friendly services - Referrals of young people to SRH services and the provision of these services - Youth-led social accountability activities to hold community and service providers accountable	

## Methodology

This mixed-methods study was part of the baseline and end-line research of the GUSO programme in Uganda. Data were collected between February and March 2017 (baseline) and September and October 2020 (end-line).

### The study context

The study was conducted in Nakigo sub-country of Iganga district (intervention area) where the GUSO programme was implemented, and Namalemba sub-country of Bugweri district (previously part of Iganga district; control area). In Iganga, 21% of the population is aged between 18 and 20 years while 57% are below 17 years [21]. In the East Central region, which Iganga and Bugweri are part of, 25% of the population is classified as poor, while 50.5% are classified as insecure non-poor [22]. Only 1% of women and 2% of men (15-49 years) had completed secondary education in 2016 [8]. Sixty percent (60%) of women and 97% of men (15-49 years) reported being employed [8]. Iganga district has a majority Muslim population [23], while there is no publicly available data on the religious composition in Bugweri district. In both district, public health facilities are most common, while there are some private health facilities. While there is a one hospital in Iganga, there are none in Bugweri [24].

### Study methods

Quantitative data were collected through a household survey in both areas, while qualitative data (through focus group discussions, in-depth interviews and key informant interviews) were collected in the intervention area.

The questionnaire (Additional file 1) was developed based on a literature review and questions from existing instruments from the Global Early Adolescent Study^2^ and the Yes I Do programme.^3^ Questions focussed on youth empowerment, knowledge and attitudes regarding SRHR, and access to SRHR information and services. The sample size was calculated to be able to detect a reduction of 10% over a 3-year period in the percentage of women who gave birth between age 15 and 24. The percentage in the East-Central region was 46.8% [25]. This provided a sample size of 425 for females (pw = 0.8; sig < 0.05; attrition rate 10%), and an overall sample size of 1700 (50% females and 50% males, and 50% intervention as well as control area) at base- and end-line. At both base- and end-line, 68 enumeration areas (EAs) were randomly selected, with probability of selection proportional to size, after which a fixed number of 25 households were randomly selected per EA by applying a fixed interval based on the listing of households.

Eight focus groups at baseline and 12 focus groups at end-line were conducted with young women and men in the age range of 15-19 years and 20-24 years, and with parents or caregivers. Seventeen in-depth interviews at baseline and 21 in-depth interviews at end-line were held with young women and men in the same age ranges, parents or caregivers, teachers, health and social workers, religious and traditional leaders, and staff of community-based organisations and youth organisations (Table 2). The focus groups and in-depth interviews captured community norms on SRHR, knowledge of and attitudes towards SRHR, young people’s ability to express themselves and their decision-making on SRHR, access to and use of SRHR information and services, and gender norms and attitudes. Eight key informant interviews at baseline and 12 key informant interviews at end-line were conducted with NGO staff and policy makers to understand their experiences with the GUSO programme and to give insight into youth SRHR. Selection of participants was purposeful and recruitment was facilitated by community health workers and GUSO partners. Topic guides are available in Additional file 2.

**Table 2:** Overview of qualitative component (only in intervention area)

Methods and participants	Baseline	End-line
**Focus group discussions**		
Girls (15-19 years)	2	2
Young women (20-24 years)	1	2
Boys (15-19 years)	1	2
Young men (20-24 years)	2	2
Parents or caregivers	2	4
**Total**	**8**	**12**
**In-depth interviews**		
Girls (15-19 years)	2	2
Young women (20-24 years)	2	3
Boys (15-19 years)	2	2
Young men (20-24 years)	1	3
Parents or caregivers	2	2
Religious and traditional leaders	2	3
Teachers	2	2
Health and social workers	3	2
CBO and youth organisation staff	1	2
**Total**	**17**	**21**
**Key informant interviews**		
NGO staff	4	5
Policy makers/ county officers	2	2
District Health Office	2	4
Law enforcement	0	1
**Total**	**8**	**12**

 Research Assistants involved in data collection, were young women and men. Involving youth in the research process has been shown to improve power asymmetries between the researcher and participant and to be successful in eliciting sensitive information [26]. Moreover, this aligned with the GUSO programme's approach of meaningful and structural involvement of young people throughout the programme [10]. Research teams were familiarised with the study objectives and trained in ethics, data collection and analysis methods. Key terms were translated to Lusoga and translated back into English to ensure fidelity to the original translations. A pre-test of all tools was conducted at both base- and end-line, after which minor refinements were made. Interviews were conducted in the language that the participant was most comfortable with (English or Lusoga). Survey data were collected using tablets, while qualitative data were digitally recorded, transcribed verbatim (and, where applicable, simultaneously translated) and randomly checked against the recordings. The field research team held daily debriefing sessions during data collection to discuss emerging issues and strategies to overcome challenges encountered, thereby optimizing sampling and data quality.

Survey data were analysed in Stata 15. The analysis consisted of descriptive and inferential statistics. A list of outcome variables is presented in Additional file 3. Key indicators that capture young people’s empowerment through the aspects of voice (self-expression) and choice (e.g., decision-making on a variety of aspects) concerning sex and relationships were selected for inferential statistics. A univariable regression analysis was conducted to assess the strength and direction of associations. This was followed by multiple variable regression analysis using the logit model, which was conducted using a difference-in-difference approach. The regression model is elaborated in Additional file 4. Odds ratios are presented. *P*-values below 0.05 are considered significant. Covariates used in the regression models were schooling status, gender, age (below 19 years and 18 years and above 18) and relationship status. These covariates were selected as they are known to have an influence over the outcome variables. Following the differences in characteristics of survey respondents concerning schooling and relationship status at base- and end-line, additional difference-in-difference analyses were conducted in disaggregated samples of in-school and out-of-school youth and single youth and youth in a relationship. Outcomes of these analyses are not presented, because they did not differ from overall difference-in-difference analysis outcomes.

Qualitative data were coded in Nvivo 12 using an inductive approach. A validation session was conducted with key stakeholders after each study phase, which further informed the findings. The use of multiple methods, respondents and the involvement of researchers from different geographical contexts and disciplines facilitated data triangulation. Narratives on young people’s sex and relationships, self-expression and decision-making were written.

### Ethical considerations

The study was approved by the AIDS Support Organisation (TASO) Research Ethics in Kampala, Uganda (TASOREC/01/17-UG-REC-009) and by the Uganda National Council for Science and Technology (SS 4221 and SS 5269) at baseline and end-line. The study was carried out in accordance with ethical guidelines of KIT Royal Tropical Institute and Makerere University. Informed written consent was given by the study participants. Consent was given by parents or caregivers for participants under the age of 18 in accordance with the above ethical guidelines.

## Results

### Respondent demographics

The baseline consisted of 1704 respondents and the end-line of 1713 respondents, spread equally between the intervention and control areas (Table 3). The male to female ratio was 56:43 at baseline and 51:49 at end-line. The age distribution was similar over time with 37% between 15 and 17 years and 63% between 18 and 24 years at baseline and 39 and 61% respectively at end-line. In the intervention area, most of the sampled youth identified as Muslim, while in the control area, identification as Muslim or Christian was more equal. The percentage of youth who reported being single was 70% in the intervention area and 66% in the control area at baseline, and 82 and 83% respectively at end-line. Similarly, the percentage of in-school youth was 37% in the intervention area and 34% in the control area at baseline, and 57 and 62% respectively at end-line. At end-line, the majority (63%) of youth in the intervention area had heard about GUSO, of which 26% had participated in the programmme. Those in the control area had also heard about it (41%), largely through the radio or schools, and of these, 12% had participated in the programme through these mediums (Table 3).

**Table 3:** Demographic characteristics

Demographic characteristics	Intervention		Control	
	Baseline	End-line	Baseline	End-line
**Gender**				
Female	515 (59.5)	447 (52)	444 (53)	431 (51)
Male	350 (40.5)	419 (48)	395 (47)	416 (49)
**Age**				
15-17	317 (37)	332 (38)	313 (37)	330 (39)
18-24	548 (63)	534 (62)	526 (63)	517 (61)
**Religion**				
Muslim	459 (53)	547 (63)	430 (51)	406 (48)
Christian	402 (46.5)	319 (37)	409 (49)	441 (52)
Other	4 (0.5)	0	0	0
**Marital Status**				
Single	603 (70)	714 (82)	557 (66)	703 (83)
Married	196 (23)	122 (14)	251 (30)	113 (13)
Divorced	28 (3)	9 (1)	19 (2)	9 (1.1)
Widowed	1 (0.1)	0	1 (0.1)	0
Living together	37 (4)	21 (2)	11 (1)	23 (3)
**Current school status**				
In school	323 (37)	495 (57)	288 (34)	524 (62)
Out-of-school	542 (63)	371 (43)	551 (66)	323 (38)
**Know about GUSO**				
Yes	NA	548 (63)	NA	348 (41)
No	NA	314 (36)	NA	496 (59)
Don’t know	NA	4 (0.5)	NA	3 (0.4)
**Participated in GUSO**				
Yes	NA	223 (26)	NA	105 (12)
No	NA	318 (37)	NA	227 (27)
I have never heard of GUSO	NA	6 (0.7)	NA	12 (1)
Don’t know	NA	1 (0.1)	NA	4 (0.5)

### Sex and relationships in Iganga: setting the scene

Seventy-six percent (76%) of the youth at baseline reported having engaged in sexual intercourse, compared to 61% at end-line (Table 4). There were no major differences between genders. This decreasing trend was statistically significant and was observed in both the intervention and control area. However, there was no significant difference in the trends between the areas. Being older than 18, a male, out-of-school and in a relationship were significantly associated with ever having sex (Table 5).

**Table 4:** Descriptive statistics

Descriptive statistics^a^	Intervention Area		Control Area	
	Baseline	End-line	Baseline	End-line
**Sex and relationships**				
**Ever had sexual intercourse**	647 (74.8)	522 (60.3)	647 (77.1)	511 (60.3)
**Self expression**				
**I am able to express my feelings about sexuality and relationships**	613 (70.9)	599 (69.2)	602 (71.8)	599 (70.7)
Ease in talking to these stakeholders about sexuality, contraception and relationships^b^				
Health workers	NA	749 (86.5)	NA	725 (85.6)
Teachers	289 (33.4)	265 (55)	476 (31.6)	487 (55.7)
Girlfriend/boyfriend/spouse/partner	717 (82.9)	700 (80.8)	707 (84.3)	653 (77.1)
Peers	646 (74.7)	648 (74.8)	548 (65.3)	644 (76)
Parents	392 (45.3)	532 (61.4)	392 (46.7)	496 (58.6)
Other family members	314 (36.3)	483 (55.8)	398 (47.4)	473 (55.8)
Religious leaders	145 (16.8)	238 (27.5)	154 (18.4)	263 (31.1)
Traditional or community chiefs	56 (6.5)	90 (10.4)	54 (6.4)	128 (15.1)
(Local) political leader	53 (6.1)	100 (11.5)	110 (13.1)	128 (13.8)
**Decision making**				
Decision making about dating and choice of partner				
**I decide for myself who to date**	787 (91)	814 (94)	760 (90.6)	785 (92.7)
**I should have the choice to decide whom to marry**	776 (89.7)	764 (88.2)	752 (89.6)	773 (91.3)
Decisions about sex				
**I feel I am able to make the decision myself if I want to have sex or not**	648 (74.9)	689 (79.6)	653 (77.8)	655 (77.3)
Decisions about contraceptive use				
**I feel confident that I can use a condom every time if I have sexual intercourse in the future**	494 (57.2)	520 (60)	452 (53.9)	480 (56.7
I find it appropriate for a boy to propose to use a condom	686 (79.3)	694 (80.1)	627 (74.7)	680 (80.3)
I find it appropriate for a girl to propose to use a condom	635 (73.5)	634 (73.2)	586 (69.8)	628 (74.1)
A couple should decide together if they want to have children	794 (91.8)	794 (91.7)	772 (92)	798 (94.2)
Men should have the final word about decisions in the household	480 (55.5)	649 (74.9)	571 (68.1)	652 (77)
I am worried about being denied access to contraceptives	450 (52)	612 (70.7)	466 (55.5)	596 (70.4)
**Currently use contraception**	328 (37.9)	314 (36.3)	334 (39.8)	252 (29.8)
Decisions about SRH services				
**Ever used SRH services**	715 (82.7)	574 (66.3)	701 (83.6)	509 (60.1)
I am worried about being denied access to SRH services	356 (41.2)	294 (33.9)	408 (48.6)	286 (33.8)
Support felt by the following people in accessing sexuality education and SRH services?^b^				
Health workers	NA	737 (85.1)	NA	736 (86.9)
Teachers	306 (35.4)	485 (56)	265 (31.6)	473 (55.8)
Girlfriend/boyfriend/spouse/partner	713 (82.4)	683 (78.9)	677 (80.7)	664 (78.4)
Peers	601 (69.5)	629 (72.6)	553 (66.9)	621 (73.3)
Parents	434 (50.2)	598 (69.1)	461 (54.9)	579 (68.4)
Other family members	324 (37.5)	490 (56.6)	420 (50.1)	494 (58.3)
Religious leaders	193 (22.3)	264 (30.5)	154 (18.4)	282 (33.3)
Traditional or community chiefs	61 (7.1)	99 (11.4)	70 (8.3)	141 (16.6)
(Local) political leader	73 (8.4)	138 (15.9)	112 (13.3)	162 (19.1)
Traditional or community chiefs	61 (7.1)	99 (11.4)	70 (8.3)	141 (16.6)
(Local) political leader	73 (8.4)	138 (15.9)	112 (13.3)	162 (19.1)

^a^Key variables in bold

^b^A scale was used where answer options included ‘very supportive, ‘supportive, ‘neutral’, ‘not so supportive, ‘not at all supportive’ and ‘not applicable’. The ‘supportive’ and ‘very supportive’ answer options are combined to present rates of support

**Table 5:** Difference-in-difference analysis on key variables - Odds ratios

Multivariable regressions	Difference-in-difference analysis odds ratios							
	Constant	Intervention*time	Intervention area	Time	Over 18 years of age	In-school	In any type of relationship	Female
I am able to express my feelings about sexuality and relationships	0.945	0.908	1.003	1.054	1.813***	0.886	1.652***	0.611***
I decide for myself who to date	3.447	1.180	1.049	1.366	2.042***	0.993	1.238	0.670**
I should have the choice to decide whom to marry	5.086	0.704	1.017	1.230	1.202	1.097	1.345**	0.979
I feel I am able to make the decision myself if I want to have sex or not	1.902	1.282	0.881	1.043	1.286**	0.984	1.557***	0.827
I feel confident that I can use a condom every time if I have sexual intercourse in the future	1.039	0.964	1.195	1.149	1.091	1.067	1.437***	0.551***
Choice to become pregnant	0.040	0.697	1.605**	1.118	3.570***	0.308**	1.736	NA
Ever used SRH services	0.304	1.270	1.071	0.291***	4.363***	0.610***	3.571***	1.208
Currently use contraception	0.057	1.354	1.000	0.771	2.651***	0.770***	4.207	0.613***
Ever had sexual intercourse	0.177	0.768	1.126	0.568***	4.861***	0.242***	13.335***	0.585***

** *p*-value > 0.05

*** *p*-value > 0.01

As explained by a key informant, those in school were less likely to have ever engaged in sex due to strict monitoring of youth’s movements by school staff. According to many adults and youth, youth in Iganga were curious to engage in relationships during puberty, often influenced by peers. Young women and men looked for various things in a relationship including sex, getting married and having a family/children. Some young people at baseline also shared that they wanted to be understood, loved and respected. At both study phases, particularly at end-line, many young women sought financial support, or items such as sanitary napkins, chapattis or Vaseline, and were willing to engage in sex in return.

“…just have to go look for a boy that will give me 5000 shillings and if he says “I love you”- you just accept because you know he will give you 5000 shillings another time so you can purchase sanitary towels or Vaseline or clothes…..” Young woman, Focus group, 15-17 years old, End-line

Young men also recognised that money played an important role for their partners. At baseline, a young man (18-24 years, focus group) explained that young men who were working used money as an incentive ‘to lure women into sex’. Over time, sex in exchange for material goods was mentioned as the dominant form of relationships for both young men and women. While men were expected to provide financially in the relationship, women were expected to be patient, loving and ‘manageable’.

### Self-expression

At both base- and end-line and in the intervention as well as the control area, approximately 70% of the young people agreed with the statement that they could ‘express their feelings about sexuality and relationships’ (Table 4). Those who were older than 18, were male and in a relationship were significantly more able to express feelings on this topic (Table 5). This was confirmed by some key informants who shared that younger adolescents found it more difficult to express themselves in relationships compared those who were older, and that gendered socialisation enabled boys and young men to better express themselves in relationships compared to girls and young women. This was also confirmed by a younger male adolescent.

Most young people considered issues related to sexuality to be sensitive and hence used metaphors to talk about this with peers, and in some cases their parents. They would approach their parents by framing their problem as one being experienced by a friend or sharing that ‘the moon was out of the sky’ which meant they were menstruating. The survey also explored whom youth felt comfortable with to speak to about sexuality, contraceptives and relationships (Table 4). At both base- and end-line, in both areas, youth found it easiest to talk to others in their age cohort, including their peers and their partner, followed by parents, other family members and teachers. Stakeholders that they found most difficult to talk to were religious, traditional and political leaders. Nevertheless, talking to teachers, parents, other family members, religious and traditional leaders seemed to have become easier over time in both areas. At end-line, a parent shared that friends were sensitizing each other on SRHR as a result of the GUSO programme’s efforts. Likewise, a key informant shared that the intervention was enabling youth to better express themselves. A key informant, two teachers and one young woman (focus group, 18-24) specifically mentioned the positive role of senior female and male teachers, who were linked to the GUSO programme.

“Yes they [referring to the GUSO programme] empower adolescents and youths to speak for themselves if something bad is happening; they encourage them to say it out”- Key Informant Interview, End-line.

While a few young participants spoke to their aunties and mothers, youth still felt some fear confiding in their parents at end-line. This was related to the norm that talking about SRHR with elders was considered shameful, and that young unmarried people, especially those under 18, should not engage in sexual relationships. On the contrary, health workers were popular confidantes among youth at end-line, and one mother specifically mentioned the GUSO health workers.

### Decision-making

At base- and end-line, young people wished to have more autonomy in their decision-making in different aspects of their life including work, schooling and relationships. At end-line, a young man (focus group, 18-24 years) expressed that those who were out of school could more easily make decisions as they needed to earn an income. The perspective of adult participants differed. At baseline, they felt that young people were not old enough to make decisions themselves and needed parental guidance, especially if parents were paying their school fees and the children were under 18 years.

“To me a child is a child, there is no decision I can leave them to make on their own. I make decisions for them because I am still giving them school fees.” - Mother, Focus group, Baseline.

At end-line, this perspective changed and parents expressed more flexibility and discussed the importance of guiding youth in decision-making. At end-line, two key informants also acknowledged the role of GUSO in enabling youth to make their own decisions.

“Right now we thank organizations like GUSO and the government via the community dialogues being organized, young people have been able to make their own decisions and as of now cases around sex have reduced.”- Key Informant, End-line.

#### Decisions about dating and choice of partner

Most youth mentioned that they would like to make their own decisions about sex, relationships and marriage at both baseline and end-line. Ninety-one percent (91%) of the youth agreed that they could decide whom to date over both study areas at baseline, which increased to 94% in the intervention area and 93% in the control area at end-line (Table 4). This represents only a marginal improvement over time. There was no significant difference between the intervention and control area over time, however, the difference over time within the intervention area was significant (Table 6). Being older than 18 years and being a male was significantly associated with being able to decide whom to date (Table 5). Youth were also asked if they felt they should be able to decide whom to marry, which most respondents agreed with. There was no significant change over time in both areas. (Table 4). Being in a relationship was significantly associated with being able to decide whom to marry (Table 4). In the qualitative interviews and focus groups, several young women explicitly expressed that it was for them to decide when and whom to marry, and if they wanted to be in a relationship, particularly at baseline.

**Table 6:** Intervention area-only regressions

Intervention area-only regressions	Constant	Time	Age	In-school	In a relationship	Gender
I am able to express my feelings about sexuality and relationships	0.919	0.957	1.932***	0.853	1.814***	0.55***
I decide for myself who to date	4.790	1.704**	1.948***	0.730	1.183	0.630
I should have the choice to decide whom to marry	3.031	0.844	1.509**	1.318	1.687**	0.932
I feel I am able to make the decision myself if I want to have sex or not	1.656	1.357**	1.297	0.930	1.525***	0.874
I feel confident that I can use a condom every time if I have sexual intercourse in the future	1.095	1.124	1.242	0.980	1.381**	0.529***
Ever had sex	0.224	0.447***	5.133***	0.206***	11.917***	0.538***
Ever used SRH services	0.352	0.376***	4.237***	0.575***	3.453***	1.230
Currently use contraception	0.067	1.070	2.936***	0.608***	3.342***	0.525***
Choice to become pregnant	0.084	0.782	2.877	0.286	2.016	NA

** *p*-value > 0.05

*** *p*-value > 0.01

At end-line, while a few youth mentioned the decision to choose their own partners, few young men also spoke about the importance of education and working (over having relationships). One young man (focus group, 15-17 years) wanted to focus on education and getting a job, while another in the same focus group felt he was too young to be in a relationship. A few youth spoke about autonomy in decisions to marry, but a male parent shared that this was a decision taken by parents after the child turned 18. Some youth and adult participants reiterated that being out of school and older could enable youth to make decisions about these topics. While the quantitative data did not confirm the role of schooling status, it did confirm that being older than 18 years was significantly associated with being able to make decisions about whom to marry, but only within the intervention area (Table 5). A few young men and women also spoke about the decision to not have multiple partners or cheat.

#### Decisions about sex

The survey data showed that 75% of young people in the intervention area and 78% in the control area were able to decide if they wanted to have sex or not at baseline (Table 4). The increase in the intervention area from 75% at baseline to 78% at end-line was significant (Table 6), while no significant change over time was observed in the control area; and the trends in both areas were not statistically different from each other (Table 5). Those who were older than 18, and those in a relationship were more likely to be able to make decisions around sex (Table 5). Although gender did not play a role as per the difference-in-difference analysis, according to a key informant and a teacher, young women were more restricted in their decision-making concerning sex and were often ‘lured by money’. A few youth at baseline also reiterated that young girls could not easily make decisions whether or not to have sex. According to a 21-year-old young man at end-line, boys did not usually refuse sex, but a girl could, if the boy did not use a condom, was not hygienic or was not able to provide money or goods.

Peers influenced young people’s decisions about whether or not to engage in sex. At both base- and end-line, young men would emulate others who had a girlfriend or had already engaged in sexual intercourse. In particular, this was perceived to be a sign of masculinity by a young woman (focus group, 15-17) at end-line, and by a key informant at baseline. More generally, it was also seen by some youth as a sign of maturity for both young women and men at end-line. Many young women engaged in transactional sex when they saw their friends with material goods they did not have. Most adult participants at end-line felt that peers were a bad influence in this regard. The influence of pornographic videos was also cited by a few youth and adult participants at end-line. Moreover, parents also mentioned discos. The descriptive statistics showed that 11% of youth in the intervention and control area at baseline had ever been physically forced to perform sexual acts without their consent. This was still reported by 11% in the intervention area at end-line, and a similar rate of 12% in the control area. Young women were more likely to experience this than young men.

#### Decisions about contraceptive use

At both base- and end-line, many young women spoke about their choice in using a condom, getting tested and opting for family planning, even if it was without the knowledge of their partner. Young men also talked about safe sex (using condoms and getting tested for STIs).

Young people were aware of a variety of contraceptive methods such as condoms, the pill and injections at both study phases. Young women and men at both study phases shared that they approached their peers for advice who informed and encouraged them about condom use. Adult participants indicated that contraceptives were commonly used by young people, at times without the awareness of their parents. At end-line, one young man (focus group, 15-17 years) shared that it was because of the GUSO programme that youth understood how to protect themselves. However, many youth and adult participants alike shared that abstinence was the first and preferred strategy within the ABC (Abstinence, be faithful, use a condom) messaging promoted by health workers, teachers and parents towards youth at both study phases.

“At school, teachers always talk to us on how to keep safe (ABC) i.e. abstinence being faithful and use of condoms.”- Young woman, 18-24, Focus group, Baseline.

“A good youth / adolescent have manners and she is in position to abstain not to indulge in sexual activities with the boys, doesn’t walk anyhow but keeps always at their home.”- Young woman, 18-24, Focus group, End-line.

Key informants shared that only abstinence-based information was allowed to be disseminated in schools. This was evident in the increasing percentage of youth who mentioned ‘abstinence’ as one of the pregnancy prevention methods over time, particularly in the intervention area, from 44 to 54%. However, the percentage who mentioned abstinence as a sole method remained steady at 10% at base- and end-line in the intervention area.^4^

At both study phases, youth particularly recognised condoms as a method to prevent pregnancy and transmission of diseases. This was also considered a reason to propose condom use between partners. At baseline, 57% of youth felt confident to propose condom use each time they had sex, in the intervention area, which increased significantly to 60% at end-line (Table 4). The control area also saw an increase, however, this was not statistically significant. There were no significant differences between the trends in the two areas over time (Table 4). Young men, and those in a relationship were more likely to be confident in proposing condom use as compared to young women, and those who were single (Table 5). Other descriptive data in the survey also indicated this gender difference. A higher percentage of youth (over 75%) in both areas at base- and end-line found it appropriate for boys to propose condom use while a lower percentage (70-75%) found it appropriate for girls to propose the same at both study phases (Table 4).

Many young people mentioned that it was possible for a girl or a boy to refuse sex if a condom was not used. However, at baseline, a young man (focus group, 18-24) mentioned that some girls believed that the condom would get stuck in them and hence refused to use them. Proposing condom use could be seen as a sign of being HIV positive or being a sex worker (‘prostitute’^5^). A parent also related a story of a couple where the young woman refused to use a condom while the young man wanted to use one, and they therefore decided not to have sex. Such stories were also related by youth at end-line. Some young men shared that if a girl wished to use a condom and the boy did not have one or did not want to, then boys might go ahead with unprotected sex. However, if the girl would still refuse, then this could lead to separation of the couple.

Although awareness of contraceptives and the ability to negotiate their use was high, access to contraceptives for young people was low. Descriptive data from the survey indicated that the percentage of young people who were worried that they would be denied access to contraceptives increased over time in both areas (Table 4). One young woman (focus group, 15-17 years) mentioned that since she had no money to buy condoms, she used the withdrawal method to avoid getting pregnant.

The percentage of youth who reported using any method of contraception was 38% in the intervention area and 40% in the control area at baseline. There was a statistically insignificant decrease to 36 and 30% respectively at end-line (Tables 4 and 5). Being male, older than 18 years and being out of school were significantly and positively associated with contraceptive use. It is worth noting that being in a relationship had no significant association with contraceptive use.

#### Decisions about SRH service use

Although many young people at base- and end-line shared that HIV could be prevented through abstinence, many other young people also found it important to get tested as well as practice safe sex. Nevertheless, a lower percentage of youth reported using SRH services over time in both areas (Table 4). The decreasing trends from 83 to 63% in the intervention area (Table 6) and 83 to 60% in the control area were significant over time. There was no significant difference between these two trends over time (Table 5). Being in a relationship, being older than 18 and being out of school were significantly associated with using SRH services (Table 5). Despite the lower percentage who used SRH services at end-line, a lower percentage worried about being denied access to services in both areas (Table 4).

Youth were also asked if they felt supported by various stakeholders around them when accessing sexuality education and SRH services (Table 4). Although the role of health workers was not investigated at baseline, they were most commonly reported to be supportive (86%) at end-line, followed by more than half of the youth feeling supported by their partners, peers, parents and other family members at both study phases. Authority figures such as religious, traditional/community and (local) political leaders were reported to be the least supportive at both study phases. There were increases over time in the levels of support that young people felt they received from all these stakeholders in both intervention and control areas, with the exception of their partners, in which there was a small decrease.

#### Decisions within relationships and marriage

Married young women (focus group, 18-24 years) at baseline expressed the importance of making their own decisions when it came to sex, choice of clothing, or moving around freely without a man or their partner. However, the descriptive statistics showed that a relatively high percentage of married youth at end-line felt that (only) men should make decisions in the household.

The survey also explored young women’s decisions about having children. There was a marginal decrease in the intervention area from 54 to 48% and an increase in the control area from 43.5 to 47% in the percentage of young women who had a child and reported that they chose to become pregnant (Table 4). These trends were neither significant over time in each area, nor between the two areas over time (Tables 5 and 6). Being out of school and being older than 18 were significantly and positively associated with choosing to become pregnant (Table 5). Descriptive statistics of survey data indicated that more than 90% of respondents at both study phases in both areas felt that a couple should decide together if they wanted to have children.

## Discussion

This study explored the changes in voice and choice concerning sex and relationships of youth in Uganda during the implementation of the GUSO programme. It is clear from the findings that there were limited changes over time in the intervention area and that this did not differ from changes in the control area. Despite the differences in schooling and relationship status between the base- and end-line sample, additional analysis indicated that both these factors did not play an influential role.

The study found that young people could express themselves regarding sexuality and relationships, but the ease of doing so depended on their age, gender and relationship status, the party they were communicating with and social norms around sexuality. The findings point to the need for the continued focus on working with young women and ensuring that young men are able to speak up to confront gender inequality, and can  be allies to women by giving them space and supporting their self-expression [3]. Communication with adults was still constrained at end-line where some youth felt fearful of confiding in their parents. This has also been found by other studies in Uganda and Sub-Saharan Africa [15, 17, 27–29] and can be linked to the unchanged (inter-generational) taboo of speaking about sexuality in the Ugandan context [30]. This taboo was evident in the use of metaphors by youth when speaking about sexuality (with their parents). It is important to move away from fear-based messaging and to improve communication between parents and youth, because this has been found to improve adolescent SRHR outcomes [31].

Youth continued to express a desire to make their own decisions around initiating sex and relationships and negotiating the space they had for this. This was influenced by attitudes of peers and parents, demographic characteristics such as age and gender, their exposure to abstinence-focussed information and gender norms. This negotiation also took place in their relationships. Our study confirms findings from other studies on transactional sex, where young women make agentic choices to engage in relationships to meet material needs [13, 29]. At the same time, young men seemed to recognise that they needed to provide for the girl to elicit her interest and maintain the relationship [29]. In a study involving adolescent girls in Western Uganda, it was found that the ‘freedom of choice and ability to negotiate’ were restricted by the exchange of material goods, due to fear of violence or an inability to ‘pay back’ the boy [29]. Although this was not investigated in detail in our study, Nabugoomu et al. (2020) cautioned that girls might feel pressured to get into such relationships to provide for their family [13]. This is concerning as our study found that the exchange of material goods for sex became the dominant form of aspirational relationships for youth over time. A recent study revealed that economic hardship associated with the COVID-19 pandemic restrictions led to an increase in transactional sex in Uganda [18]. These findings also point to the need for more interventions that aim to transform social and gender norms around sexuality, which Malhotra et al. (2019) propose in combination with interventions addressing structural drivers such as access to health and education services, laws and policies among others [32]. The recent challenges in implementing CSE and the increasing restrictive environment in Uganda may explain the emphasis on abstinence by both adults and youth and in relation to this, the decrease in sexual activity among survey respondents observed in this study. The Ugandan government moved from an open stance on sexual health to conservative beliefs, influenced by US-funded abstinence-only initiatives such as PIASCY (Presidential Initiative on AIDS Strategy to Youth) and a growing movement of Christianity which frames Christian beliefs as ‘local’ [12]. As Moore et al. (2021) put it- “the Museveni government’s initial ‘ABC’ approach lost its ‘C’” [12]. Religious leaders are powerful players in this landscape, as the Inter-Religious Council is responsible for oversight of most educational institutions in Uganda [12]. Following the ban on CSE in 2016, GUSO programme staff used the PIASCY guidelines, which compromised the quality of education due to its primary focus on HIV/AIDS and abstinence [33]. The new guidelines for sexuality education, released in 2018, retained a focus on abstinence and were accompanied by increased bureaucratic hurdles and monitoring by the government (personal communication, 2021; [12]). Although societal norms in Uganda dictated a preference for abstinence even prior to the CSE ban [34], these contextual changes are crucial to frame the results of this study. For instance, the lower uptake of SRH services over time may be due to the lack of CSE which has shown to be effective in creating demand for SRH services [35]. Such education would need to be linked to referrals to health centres, which have also shown promise in increasing uptake [36, 37]**.** While the GUSO programme had a focus on improving the policy environment, a large part of the activities were geared towards changes at the individual and community level [20] (See Table 1). The impact of these efforts may have been eclipsed by changes at the policy level. COVID-19 has also reduced the access to SRHR information and services in Uganda [18, 38] and may partially explain the decreasing percentages of SRH service and contraception use over time as found in this study.

These contextual changes help in understanding the limited changes made by the GUSO programme in young people’s voice and choice concerning sex and relationships, as they reinforce existing unequal gender norms, economic hardship and a taboo on youth sexuality.

Challenges in implementing multicomponent programmes as well as the difficulty in measuring the effect of such programmes have been documented before. The multicomponent approach has been hailed widely in recent years, most importantly by the Lancet which outlines “the most effective actions for adolescent health and wellbeing are inter-sectoral and multicomponent” [39]. However, a commentary on what works for SRHR interventions also revealed that many such programmes were implemented in a piecemeal approach in reality and in a ‘low dosage’ [40]. A review on child marriage interventions highlighted challenges of the multicomponent approach, such as the setting up and delivery of holistic programmes in short time spans and lower uptake by girls and families due to high time commitments. This review found single-component interventions to be more scalable and sustainable for these very reasons [41]. This study used a quasi-experimental research design due to limitations of using experimental designs which have been widely discussed elsewhere [42, 43]. There is a need to combine this with process evaluations, including qualitative research methods, which focus on the development and implementation of initiatives and explain the ‘how’ and ‘why’ [44]. Adding a qualitative study component in the control area could have helped to explain some of the positive changes observed there in this study. These changes could have been a result of an increasing number of organisations working on SRHR [45, 46] and of the wide coverage of the GUSO programme wherein youth from the control area attended schools based in the intervention area in which the GUSO activities were implemented. Lastly, the need to have more contextually-driven studies remains relevant in evaluating such large scale programmes.

## Conclusion

The limited effect of the GUSO programme on increasing young people’s voice and choice concerning sex and relationships in Uganda can be understood in the context of a ban on CSE and more recently the COVID-19 pandemic. These structural and emerging contextual factors enforce the taboos and norms around youth sexuality, particularly women’s sexuality, and hinder young people’s access to SRHR information and services. These findings call for continued efforts of multicomponent and more targeted programmes that expand on creating an enabling and supportive environment for young people’s empowerment concerning their SRHR.

## Supplementary Information

**Additional file 1.** Structured questionnaire (with Lusoga translations).

**Additional file 2.** Topic guide for focus group discussions (FGDs) with young girls and boys (15-24 years old) with Lusoga translations.

**Additional file 3.** Description of outcome variables.

**Additional file 4.** The Regression Model.

## Data Availability

The datasets generated and analysed during the current study are available from the corresponding author on reasonable request.

## Abbreviations

CSE: Comprehensive Sexuality Education
GUSO: Get Up Speak Out
HIV: Human Immunodeficiency Virus
ICPD: International Conference of Population and Development
PIASCY: Presidential Initiative on AIDS Strategy for Communication to Youth
SRH: Sexual and Reproductive Health
SRHR: Sexual and Reproductive Health and Rights
STI: Sexually Transmitted Infections

## Footnotes

1 Straight Talk Foundation (STF); Restless Development (RD); National Forum of People Living with HIV/AIDS Networks in Uganda (NAFOPHANU); Reach a Hand Uganda (RAHU); Uganda Network of Young People Living with HIV/AIDS (UNYPA); Family Life Education Program (FLEP); Reproductive Health Uganda (RHU); and Centre for Health, Human Rights and Development (CEHURD).

2 https://www.geastudy.org/

3 https://www.kit.nl/project/yes-i-do/

4 In the intervention area, 44% of youth at baseline mentioned abstinence as one of the pregnancy prevention methods, and 10% mentioned it as the sole method. This increased to 54% and remained steady at 10% respectively. In the control area, this was 42 and 10% at baseline, which increased to 46% and decreased to 7% at end-line.

5 Prostitute—as mentioned in an interview with a key informant, HIV positive- according to field notes.
