# Supplementary material for: Young people’s choice and voice concerning sex and relationships: effects of the multicomponent Get Up Speak Out! Programme in Iganga, Uganda
Source: BMC Public Health. 2022 Aug 23;22:1603. doi: 10.1186/s12889-022-13919-x (PMC9396562; doi:10.1186/s12889-022-13919-x)
Supplement: Supplementary file 1 — Additional file 1. Structured questionnaire (with Lusoga translations). [file 12889_2022_13919_MOESM1_ESM.pdf]

Additional File 1

Structured questionnaire (with Lusoga translations)

Question number	Question text	Type of question	Choices	Hint
0.00	Section 0 - General information			
0.01	Please register the location			
0.02	In which sub-county are you located?/ Lino eigombolola balyetaki		Nakigo	
			Namalemba	
0.03	In which enumeration area are you located?/ Wano beetawo wa?	integer		
0.04	Ask for consent to take part in the interview		Consent	Find out the age of the prospective respondent. Please explain the objective and consequences of participation to this survey to the respondent (refer to the consent/assent form). If minor, obtain consent from the main caregiver/ guardian of the family, prior to seeking their consent. Use the consent form. The respondents should sign. If uncomfortable, verbal consent is permissble.
			Refused	

	Thank you for agreeing to participate! This survey will take you through different sections where we invite you share your knowledge and opinions related to sexuality and sexual & reproductive health rights, which we refer to as SRHR./ Weebale inho okwikiriza okuba omu kwaabo abegaise mu kubuliriza kuno. Okubururirza kulime ebitundu nga ebiwera. Oidha kutukobera byoidhi ni byolowooza kubigema ku byomukwaano, okwegaita, okuzaala, enkolagana ya abakazi/abawala na basadha/abalenzi, neidembe lyo kubigema kweebyo.			
0.05	Why did the person refuse to participate?/ Kiki ekikulobeire okwegaita mu kunonereza kuno?	text		
0.06	Interviewers ID	integer 1-12		
1	Section 1 - Respondents background			
1.01	What is your gender?/ Oliki ekikula kyo?	single response	Female/ muwala/mukazi	Observe. If not sure - ask
			Male/ mulenzi/musadha	
1.02	Can you tell me your age (in numbers)?/ Olina emyaaka emeka?	integer		If don't know, ask for year and calculate age.
1.03	Do you have a national ID card or birth registration card?/ Olina endaga muntu eya Uganda or ekaadi elaga lwewazaalibwa?	single response	Yes/ yi	
			No /Mbe	
			Don't know/tidhi	
			No Answer/taizemu	
1.04	What is your religion?/ Oliwaidiini ki?	single response	Catholics/mukatuliki	
			Protestants/ mukuristaayo	
			Born again/ mulokole	
			Other Christian groups/ abakuristaayo abandi	
			Muslim/ musilaamu	
			Other - specify/ erindi …………………………….	
			None/ azira idhini	
1.05	What is your ethnicity?/ Oliwaigwanga ki?	single response	Musoga	
			Other - specify/ abandi wandiika ………..	
1.06	What is your marital status?/ Olimufumbo? Wafumbirwaku?	single response	Single (never married)/ Tifumbirwangaku	

			Married/ mufumbo	
			Divorced/ separated/ twayawukana	
			Widowed/ Na/semwandu	
			Living together/ tubawalala aye tituli bafumbo	
1.07	If single/ divorced/ widowed (1,06) Do you have a partner?/ Olinayo mukwaano gwo?	single response	Has a boyfriend/ ninayo muganzi wange omulenzi	
			Has a girlfriend/ ninayo muganzi wange	
			Does not have a partner/ nzira muganzi	
1.08	Who do you live with?/ Oba naani?	multiple response	Mother & Father/ maama ni baaba	You may mark more than 1 option
			Mother/ maama	
			Father/ baaba	
			Mother in law/ Inhazaala wange	
			Father in law/ sezaala wange	
			Grandmother / daada omukazi	
			Grandfather/ daada omusadha	
			Aunt/ ssenga	
			Uncle/ kooja	
			Sister(s)/ muganda wange omukazi	
			Brother(s)/ muganda wange omusadha	
			With husband/ wife/ omwaami oba omukwaala	
			Alone/ zzenka	
			Children/ abaana	
			Other - specify/ owundi - wandiika………	
1.09	What is the total size of your household?/ Muli bameka waka nga musula wano era nga muliira walala?	integer		This should be the total number of people who eat together/ live together as a family unit. Write down "1" if respondent lives alone.
1.10	What is the highest level of education that you obtained so far? (finished)./ Wasoma kutuuka mukibiina ki?	single response	Primary education/ primary	Please mark only one option
			O level/secondary	

			A level/higher<br>Tertiary education (institutions other than university after O/A level)/ Amasomero agemirimo ngomaze secondary oba higher<br>University/ universite<br>Not finished any of these levels (no formal education)/tisomangaku	
1.11	Are you currently in school?/ Osoma?	single response	Yes/ Yi<br>No/ mbe	
1.12	If yes (1,11), what is the level of education you are currently studying?/ Olimukibiina kyakumeka?	single response	Primary education/ primary<br>O level/siniya, secondary<br>A level/higher<br>Tertiary education (institutions other than university after A level)/Amasomero agemirimo ngomaze secondary oba higher<br>University/ universite	Please mark only one option
1.13	Have you ever dropped out of school?/Wavaaku mwisomero nga tosoma?	single response	Yes/ yi<br>No/ mbe	
1.14	If yes (1,13) What was the reason for dropping out of school?/ Kiki ekyakutoola muisomero?	multiple response	Lack of fees/ materials/feezi oba ebyokukozesa byabula<br>Domestic/ family responsibility/ nalina okukola emirimu jawaka<br>Illness/ nali mulwaire<br>Parents died while still young/ abazaile baafa<br>Pregnancy/ got a child/ nafuna enda oba nazaala<br>School too far/ not accessible/ eisomero lyaali wala<br>No time/ nali nzira biseera<br>Did not like school/nali tyenda isomero<br>Got married/ nafumbirwa<br>Suspended/ expelled/bangoba kwisomero	Do not read the list, tick all that apply

			Feared discrimination/ stigma/ natya okusosolebwa<br>Don’t remember/tidukira<br>No Answer/taizemu<br>Other - specify/ ekindi …………………………….	
1.15	What level of education did your mother attain?/ Maama wo yakoma mukibiina ki? Yasomayo koosi?	single response	No education/Tiyasomaku<br>Primary education/ primary<br>O level/siniya, secondary<br>A level/higher<br>Tertiary education (institutions other than university after A level)/Amasomero agemirimo ngomaze secondary oba higher<br>University/ universite<br>Other - specify/ekindi ……………………….<br>I don't know/tidhi	
1.16	(If any type of education 1,15) How many years of education did she complete?/ Okutwalira awalala, mwisomero yamalamu myaaka emeka?	text		Number of years starting from primary school. IF RESPONDENT DOES NOT KNOW TYPE 'DK'
1.17	What level of education did your father attain?/ Baabawo yakoma mukibiina ki? Yasomayo koosi?	single response	No education/Tiyasomaku<br>Primary education/ primary<br>O level/siniya, secondary<br>A level/higher<br>Tertiary education (institutions other than university after A level)/Amasomero agemirimo ngomaze secondary oba higher<br>Other - specify/ekindi ……………………….<br>I don't know/tidhi	
1.18	(If any type of education 1,17) How many years of education did he complete?/ Okutwalira awalala, mwisomero yamalamu myaaka emeka?	text		Number of years starting from primary school. IF RESPONDENT DOES NOT KNOW TYPE 'DK'

1.19	Do you have a job/ are you involved in labour?/ Okola? Okukola kutalira mu okuba omukyaala waka, okwekozesa no kukola mubifo ebitali bya ofiisi.	single response	Yes/ Yi<br><br>No/ mbe	Labour includes housewife, informal work and self employment.
1.20	If yes (1,19) What type of work do you do?/ Okola mulimu ki?	single response	Unpaid work (e.g.homemaker/housewife)/tisasulwa, ind mukyaala waka.<br>Subsistence farming/nima byakulya, tikusuubula<br>Informal trading/ nsubula katonotono<br>Casual, daily labour/ mpakasa leda leda<br>Contract work/ nkozesebwa ebisera ebindi<br>Self-employed/nekozesa mwene<br>Part-time permanent employment salaried/Ndimukozi wamusaala aye tikola lunaku oba naku donadona.<br>Full-time permanent salaried employment/ ndimukozi wamusaala era nkola omwezi gwonogwona.<br>Other - specify/ekindi ……………………….	Fill in main job
1.21	Who is the primary/main income-earner in your household?/ Wano waka ewaimwe, ani asinga kulabilira maka mubyenfuna?	single response	Father/ baaba<br><br>Mother/ maama<br>Grandfather/ daada omusadha<br>Grandmother/ daada omukazi<br>Uncle/ kooja<br>Aunt/ ssenga<br>Brother(s)/ muganda wange omusadha<br>Sister(s)/ muganda wange omukazi<br>Husband/ wife/ omwaami, omukyaala<br>Myself/ nze mweene<br>Other - specify/owundi ……………….	Select one

1.22	How much money (income) comes into your household each month (on average)?/ Bwogerageranya, mu mwezi, amaka gano okutwalira awalala, gaingiza sente nga imeka?	text		IF RESPONDENT DOES NOT KNOW TYPE 'DK'
2.00	Section 2 - SRHR information and education. In this section, we wish to understand if you know of any information or education regarding sexuality and sexual & reproductive health and rights./<br>Mukitundu ekiiraku twenda kutegeera oba oidhi, wategezebwa oba wasomesebwaku kubigema kubyomukwaano, okwegaita, okuzaala, enkolagana ya abakazi/abawala na basadha/abalenzi, neidembe lyo kubigema kweebyo.			
2.01	Have you ever received/ acquired information about sexuality or sexual and reproductive health?/ Wafunaku okutegezebwa (ebyokusoma, amawulire) kwona kwona kubyomukwaano, okwegaita, okuzaala, enkolagana ya abakazi/abawala na basadha/abalenzi, neidembe lyo kubigema kweebyo?	single response	Yes/ Yi	
			No/ mbe	
2.02	If no (2,01) Why did you never receive information about sexuality or sexual and reproductive health?/ Lwaaki tofunanga kutegezebwa ku kubyomukwaano, okwegaita, okuzaala, enkolagana ya abakazi/abawala na basadha/abalenzi, neidembe lyo kubigema kweebyo?	multiple response	Don’t need the information/ amawulire tigetaaga	You may mark more than one option
			Don’t know where to get it / tidhi waafuna ago amawulire	
			Too afraid to ask for information/ ntya okubisa	
			Not available/ago amawulire gazirawo	
			Other - specify / ekindi wandiika ………	
2.03	If yes (2,01) From whom (which person) did you receive information about sexuality or sexual and reproductive health?/ Aani eyakutegeza oba eyakuwa amawulire agagema kwebyo?	multiple response	Parents/ abazaile	You may mark more than one option
			Other family members/ abantu abandi abawaka	
			Religious leader/ abakulu abeidini	
			Health provider/ abasawo	
			Friends/emikwaano	
			Peer educator/ counsellor/ banange bwetwagayaga	

			Youth club/ ekibiina kyabavubuka	
			Teacher/omusomesa	
			Traditional leader/omusawo wekinansi	
			Media (TV, radio, newspaper/ magazine)/ empulizaganya nga television, radio, amawulire no butabo	
			Social media and internet/ omutimbagano gwa internet neisimu	
			Other - specify/ ekindi ……………………………	
2.04	If yes (2,01) Was it easy to get this information?/ Kyaali kyangu okufuna amawulire oba okutegezebwa okwo?	single response	Easy/ kyangu	
			Difficult/ kizibu	
			Very difficult/kizibu inho	
2.05	If yes (2,01) Did you find the information beneficial?/ Bwebakukobera, bwewasoma oba bwewawulira bwaakuyamba?	single response	Yes/ Yi	
			No/ mbe	
			Don't know/ tidhi	
2.06	If no (2,05) Why was the information not beneficial?/ Lwaaki tibyakuyamba?	multiple response	The information did not meet my needs / byebankobera oba byenafuna tibyanamba	You may mark more than one option
			I was not comfortable / tyawulira idhembe	
			Did not understand the information /bwebankobera oba amawulire gebampa tyaagategera	
			Other - specify/ ekindi ……………………………	
2.07	If yes (2,05) What information did you find most beneficial?/ Kubyebakukobera, bwewasoma oba bwewawulira, biki ebyasinga okukuyamba?	multiple response	Where to access SRH Services/ ewokwaagana empereza denetaaga	Don't read the list and mark what is said. You may mark more than one option.
			Different methods of Family Planning/Contraception / ebyokukozeza ebyendawulo okuziyiza okufuna enda	
			HIV/ STI Testing / okwebeza siliimu	
			Information regarding puberty / ebigema ku buvubuka	

			Information regarding sexual relationships and love/ebigema ku byokwegaita nomukwaano	
			Other - specify/ ekindi …………………………..	
2.07	If yes (2,05) Why was the information beneficial?/ Lwaaki byakuyamba?	multiple response	The information met my needs/ I can use the information in my daily life/ amawulire genafuna ganamba okutuukiriza byenetaaga, nsobola okubikozesa bulidho.	You may mark more than one option
			The information was easy to understand / amawulire gaali mangu okutegeera	
			The provider made me feel comfortable/ eyali atuuwereza yanamba okuba neidembe oba emirembe	
			Other - specify/ ekindi …………………………..	
2.08	(If ever been to school) Have you ever received education about sexuality or sexual and reproductive health in school?/ Wasomeebwaaku kubigema kubyomukwaano, okwegaita, okuzaala, enkolagana ya abakazi/abawala na basadha/abalenzi, neidembe lyo kubigema kweebyo mwisomero?	single response	Yes/ Yi	
			No/ mbe	
2.09	If no (2,08) Why did you never receive sexuality education in school?/ Lwaaki tiwasomesebwa?	single response	My school doesn't provide it/ eisomero lyange tilibisomesa	
			We have not reached the subject yet/ tukaali kubituukaku	
			Don't know/ tidhi	
			Other - specify / ekindi wandiika ………	
2.10	If yes (2,08) Who provided the sexuality education in school?/ Ani eyakusomesa?	multiple response	Teacher/omusomesa	You may mark more than one option
			Friends/emikwaano	
			Peer educator/ banange bwetwagayaga	
			NGO/ ebibiina ebyanakyeewa	
			Health worker / omusawo	
			Other - specify/ owundi - wandiika………	
2.11	If yes (2,08) Did you find the sexuality education in school beneficial?/ Byebaakusomesa byakuyamba?	single response	Yes/ Yi	
			No/ mbe	

2.12	If no (2,11) Why was the sexuality education in school not beneficial?/ Lwaaki tibyakuyamba?	single response	The information did not meet my needs/ I cannot use it in my daily life/ byebankobera oba okumpa tibyanamba, tibikozesa<br>I was not comfortable/ tyawulira mirembe<br>The person providing the education was not comfortable/ eyali alikutwegeresa yali azira idembe oba mirembe<br>Did not understand the information /bwebankobera oba amawulire gebampa tyaagategera<br>Other - specify/ ekindi …………………………….	
2.13	If yes (2,11) What aspects of sexuality education in school did you find most beneficial?/ Ku byebaakusomesa, biki ebwakuyamba?	text	Where to access SRH Services/awokufuna empeereza ku kubyomukwaano, okwegaita, okuzaala, enkolagana ya abakazi/abawala na basadha/abalenzi, neidembe lyo kubigema kweebyo mwisomero<br>Different methods of Family Planning/Contraception/ Engeri endendawulo odokweziyiza okufuna enda<br>HIV/ STI Testing/ okwekebeza siliimu<br>Information regarding puberty / ebigema ku buvubuka<br>Information regarding sexual relationships and love/ebigema ku byokwegaita nomukwaano<br>Other - specify/ ekindi …………………………….	Don't read the list and mark what is said. You may mark more than one option.
2.13	If yes (2,11) Why was the sexuality education beneficial?/ Lwaaki byakuyamba?	multiple response	The information met my needs/ I can use the information in my daily life/ amawulire genafuna ganamba okutuukiriza byenetaaga, nsobola okubikozesa bulidho.<br>The information was easy to understand / amawulire gaali mangu okutegeera<br>The provider made me feel comfortable/ eyali atuuwereza yanamba okuba neidembe oba emirembe<br>Other - specify/ ekindi …………………………….	You may mark more than one option

2.14	If yes (2,01) or (2,08) When you received sexuality-related information or education, were you advised where to access sexual and reproductive health services, in case you need them?/ Bwe wasomesebwa ni bwe wategezebwa ku bigema kubyomukwaano, okwegaita, okuzaala, enkolagana ya abakazi/abawala na basadha/abalenzi, neidembe lyo kubigema kweebyo, walagirirwa yoyinza okufuna obuyambi oba ebyo kukozesa bwobanga obyetaaze?	single response	Yes/ Yi	
			No/ mbe	
			Don't remember/ tidukira	
2.15	If yes (2,14) When you were advised where to access services, who advised you?/ Ani eyakulagirira oba eyakuwa amagezi?	multiple response	Friends/emikwaano	You may mark more than one option
			Peer educator/ banange bwetwaagayaga	
			Teacher/omusomesa	
			Parents/ abazaile	
			School nurse/ counsellor/ Omusawo owokwisomero, abudha budha	
			Health worker / omusawo	
			NGO/ ebibiina ebyanakyeewa	
			Mobile platform/ eisumu - whatsup nebindi	
			Online/internet/ kumutimbagano gwainternet	
			Other - specify/ owundi - wandiika………	
2.16	From whom do you prefer to receive information and education about sexuality or sexual and reproductive health?/ Ani gwewandiyenze okukiteegeza oba okukusomesa kubigema ku byomukwaano, okwegaita, okuzaala, enkolagana ya abakazi/abawala na basadha/abalenzi, neidembe lyo kubigema kweebyo?	multiple response	Friends/emikwaano	Do not read list. Tick all that apply.
			Peer educator/ banange bwetwaagayaga	
			Teacher/omusomesa	
			Parents/ abazaile	
			Other family members/ abantu abandi abawaka	
			Health facility/ eidwaliro	

			Mobile platform/ eisumu - whatsup nebindi	
			Online/internet/ kumutimbagano gwainternet	
			Youth club/ ekibiina kyabavubuka	
			Church /mosque / amasinzizo, Kanisa oba omusikiti	
			TV/ television	
			Radio	
			Other - specify/ ewandi wandika ………	
3.00	Section 3 - SRHR Services - access and use: In this section, we invite you to share your access and use of SRHR services. Butti, twendha otukobere kubigema ku kufuna nokukoseza ebyetagiza okukozesebwa ku bigema ku byomukwaano, okwegaita, okuzaala, enkolagana ya abakazi/abawala na basadha/abalenzi, neidembe lyo kubigema kweebyo./			
3.01	Do you know of any place in the community which provides sexual and reproductive health services? Oidhiku awantu wona wona mukitundu kino omuntu wayinza okuwerezebwa kubigema ku byomukwaano, okwegaita, okuzaala, enkolagana ya abakazi/abawala na basadha/abalenzi, neidembe lyo kubigema kweebyo	single response	Yes/ Yi	
			No/ mbe	
			Don't know/ tidhi	
3.02	If yes (3.01) What services are provided at this place?/ Buwerezaki bwebalinabwo oba bwebagaba?	multiple response	Antenatal & postnatal services/ obwitandabi ngo okaali ni nga omaze okuzaala	Do not read list. Tick all that apply.
			Family planning services/ okutegeka amaka nga okozesa okweziyiza okufuna enda nebindi	
			VCT/ okubudhabudha nokukeberwa siliimu	
			STI testing / okukeberebwa obulwaire obwobukaba	
			PMTCT/ okuziyiza maama obutagemya mwaana siliimu nga akaali kuzaalibwa, neera nga amaze okuzaalibwa	
			Post abortion care/ obwidandabi nga enda eviiremu oba nga etooleibwamu	

			Life skills and sexuality counselling/ obwegerese kungeri omuntu gyasobola okubaawo obulungi nokubudhabudhibwa kubigema ku byomukwaano, okwegaita, okuzaala, enkolagana ya abakazi/abawala na basadha/abalenzi.	
			Counseling for sexual violence/ okubudhabudhibwa ku kukakibwa omukwaano	
			Child protection services/ Empereza kukukuuma abaana nga bali bulungi	
			Hotlines/ amasimu goyinza okukubaku nga olina ekizibu	
			Other - specify/ ekindi …………………………….	
3.03	How can pregnancy be prevented?/ Omuntu ayinza atya okweziyiza okufuna endha?	multiple response	Abstinence/ obutegaita na muntu	Do not read the list, tick which ones are said
			Male condom/ akapiira ka galimpitawa aka basadha	
			Female condom/ akapiira ka galimpitawa akabakazi	
			Contraceptive pill/ amakerenda agaziyiza okufuna enda	
			Injections/ empiso	
			Implant/ akakerenda kebata mumubiri	
			IUD/ akawera kebata munda mubukyaala	
			Sterilization/ okukomya okuzaala	
			Morning after pill/ emergency pill/ Eikerenda omukazi lyamira nga yakamala okuba nomusadha	
			Withdrawal/okumalira kiliya	
			Observing safe days/ okubala enaaku notegeera enaku dotasobola kufuna nda	
			Don't know/ tidhi	
			No Answer/taizemu	
			Other - specify/ ekindi …………………………….	

3.04	Whose responsibility is it to prevent pregnancy?/ Aani avunaanizibwa okubona nti omuwala/omukazi tavuna ndha?	single response	Boy / man/ mulenzi, musadha	
			Girl/ woman/ muwala, mukazi	
			Both/ bombi	
3.05	Have you ever engaged in sexual intercourse?/ Wegaitaku no muntu yena yena?		Yes/ Yi	
			No/ mbe	
3.06	If yes (3,05), at age did you start having sexual intercourse?/ Olwasooka, walinemyaaka emeka?	integer		
3.07	Do you currently use any contraception?/ Eriyo kyokozesa okweziyiza okufuna enda?	single response	Yes/ Yi	
			No/ mbe	
3.08	If no (3,07) Why do you not use any contraception?/ Lwaaki?	multiple response	Never thought about it/ tikilowoozangaku	Do not read the list, tick which ones are said
			My spouse/ partner/ boyfriend/ girlfriend disapproves/ munange taikiriza	
			I am worried about the side-effects/ nerarikira obulabe obubirimu	
			I want (more) children/ nenda kuzaala baana	
			I don't agree with contraception/ tiikirikiza mu kweziyiza kufuna nda	
			It makes me uncomfortable during sex/ bimbuza emirembe bwembanga negaita	
			I did not manage to ask/ negotiate the use of a contraceptive with my partner/ tyasobola kusaba oba okwikirizaganya nimunange kubyokweziyiza okufuna enda	
			It reduces sexual satifaction/ desire/ kukendeeza okumatizibwa mukwegaita	
			I did not know about contraceptives/ ebyokweziyiza okufuna enda tyabumanaaku	
			No method available at the time/nzira kyensobola kukozesa buti	

			I do not know where to go to get contraceptives/ tiidi waakufuna ebiziyiza kufuna nda	
			Not sexually active/ nzira musadha, tyegaita	
			Refused to obtain contraceptives by health care provider(s)/ Abasawo baaloba okumpa ebiziyiza kufuna nda	
			Other - specify/ ekindi …………………………….	
3.09	If no (3,07) So you do not use contraception, but would you liked to use it now?/ To kosesa kintu kyona kyona kweziyiza kufuna ndha; aye wandiyenze okufuna kyokozesa?	single response	Yes/ Yi	
			No/ mbe	
3.10	If yes (3,07) What method of contraception do you use?/ Kiki kyewandiyenze okukozesa?	multiple response	Abstinence/ obutegaita na muntu	Do not read the list, tick which ones are said
			Male condom/ akapiira ka galimpitawa aka basadha	
			Female condom/ akapiira ka galimpitawa akabakazi	
			Contraceptive pill/ amakerenda agaziyiza okufuna enda	
			Injections/ empiso	
			Implant/ akakerenda kebata mumubiri	
			IUD/ akawera kebata munda mubukyaala	
			Sterilization/ okukomya okuzaala	
			Morning after pill/ emergency pill/ Eikerenda omukazi lyamira nga yakamala okuba nomusadha	
			Withdrawal/okumalira kiliya	
			Observing safe days/ okubala enaaku notegeera enaku dotasobola kufuna nda	
			Other - specify/ ekindi …………………………….	
3.11	If yes (3,07) Who provided you the contaceptive method?/ Aani eyakuwa oba wabitoolawa?	multple response	Peer educator/ banange bwetwagaya	You may mark more than one option
			Health facility, medical staff/ eidwaliro oba abasawo	
			School/ kuisomero	

			Youth club/ ekibiina kyabavubuka	
			Community health worker/ abasawo abakola, abatambula mukitundu	
			Outreach services (open day/ routine services)/ abantu abaidha okuwereeza mukitundu	
			Pharmacies/ shops/ amaduuka agatunda obulezi	
			Other/ owundi ……………………………………	
3.12	(For female) Have you ever been pregnant?/ Wafunaku endha?	single response	Yes/ Yi	
			No/ mbe	
3.13	If yes (3,12) Was it your choice to become pregnant?/ Niwe eyesalawo nti ogifune? Wali ogyendha?	single response	Yes/ Yi	
			No/ mbe	
3.14	If yes (3,12) How old were you at your first pregnancy? Kweeyo endha eyasooka, walina emyaaka emeka?/	integer		
3.15	How many children do you have? Olina abaana bameka?	integer		If no children, write 0
3.16	If children (3,15) (For male) At what age did you become a father?/ Wafuuka baaba nga olina emyaaka emeka?	integer		
3.17	If children (3,15) Did you want to become a father at this time? (For male)./ Olwo olwasooka, wali oyenda okufuuka baaba?	single response	Yes/ Yi	
			No/ mbe	
3.18	Which of the following services have you used?/ Waliwo empereza oba oyambibwa okuwera kubigema ku byomukwaano, okwegaita, okuzaala, enkolagana ya abakazi/abawala na basadha/abalenzi, neidembe lyo kubigema kweebyo. Nkobera oba wakozesaku bino:	multiple response	Antenatal & postnatal services/ obwitandabi ngo okaali ni nga omaze okuzaala	Read all and tick which ones used
			Family planning services/ okutegeka amaka nga okozesa okweziyiza okufuna enda nebindi	
			VCT/ okubudhabudha nokukeberwa siliimu	
			STI testing / okukeberebwa obulwaire obwobukaba	
			PMTCT/ okuziyiza maama obutagemya mwaana siliimu nga akaali kuzaalibwa, neera nga amaze okuzaalibwa	

			(Post) abortion care/obwidandabi nga enda eviiremu oba nga etooleibwamu<br><br>Life skills and sexuality counselling/ obwegerese kungeri omuntu gyasobola okubaawo obulungi nokubudhabudhibwa kubigema ku byomukwaano, okwegaita, okuzaala, enkolagana ya abakazi/abawala na basadha/abalenzi.<br>Child protection services/ Empereza kukukuuma abaana nga bali bulungi<br><br>Hotlines/ amasimu goyinza okukubaku nga olina ekizibu<br>Counseling for sexual violence/ okubudhabudhibwa ku kukakibwa omukwaano<br><br>Haven't used any service/ ezira kyenakozesaaku	
3.19	If not used any service (3,18) Why havent you used any of the services?/ Lwaaki? tobikozesangaku?	multiple response	I don't know about any such services/ ezira mpereza gyendidi<br>I never had the need to go/ tibyetaagangaku<br><br>My parents disapprove/ abazaile tibandikiriza<br><br>My spouse/ partner/ boyfriend/ girlfriend disapproves/ munange taikiriza<br><br>I am afraid to be scolded at the health facility/ tya nti kwiidwaliro baida kunvuma<br><br>It costs too much / sente ningiinho<br><br>Its too far away/ wala inho<br><br>I am shy/ ninensoni<br><br>Not available in my community/ byo mukitundu kyaife biziramu<br><br>Never thought about it/ tikirowoozangaku<br><br>Other - specify/ ekindi ……………………………	You may mark more than one option
3.20	If you have used these services (3,18), who provided them?/ Okuwerezebwa kuno wakwaagana wa? Ani eyakuweereza?	multiple response	Peer educator / banange bwetwaagayaga	You may mark more than one option

			Health facility, medical staff/ eidwaliro oba abasawo	
			School/ kuisomero	
			Youth club/ ekibiina kyabavubuka	
			Community health worker/ abasawo abakola, abatambula mukitundu	
			Outreach services (open day/ routine services)/ abantu abaidha okuwereeza mukitundu	
			Pharmacies/ shops/ amaduuka agatunda obulezi	
			Private clinic/ amadwaliro agaabantu	
			Other - specify/ ewandi wandika ………	
3.21	If yes to VCT/STI testing/ PMCTC (3,18) Have you had an STI in the last 12 months?/ Walwaalaku obulwaire obugema kukwegaita (obwabukaba) mu mweezi 12 egibise?	single response	Yes/ Yi	
			No/ mbe	
3.24	The last time you used any of these services (3,18) How did you find the quality? Please rate on a scale of 1 -5 with 1 as lowest quality support and 5 as highest quality. Lwewasemba okukoseza kumpereza edho, empereza yene yali etya?/ Bwetuba tugaba makisi, kwitaanu obawaaku imeka?	single response	Very low/ bad quality/ mbi inho	
			Low/ bad quality/ mbi	
			Average quality/ eri wagati awo	
			Good quality/ nungi	
			Excellent quality/ nungi inho, mbitirivu	
3.25	The last time you used SRHR services or contraception (3,07 or 3,18), did someone refer you to go there?/ Lwewasemba okukoseza kumpereza edho, eriyo eyakukoba okujaayo?	single response	Yes/ Yi	
			No/ mbe	
			Don't remember/ tidukira	
3.26	If yes (3,25) Who referred you?/ aani eyakukoba okujaayo?	single response	Friends/emikwaano	Do not read, tick which one is said
			Peer educator/ banange bwetwagaya	
			Teacher/ abasomesa	

			Parents/ abazaile<br>School nurse/ counsellor/ Omusawo owokwisomero, abudha budha<br>Health worker/ omusawo<br>NGO/ ebibiina ebyanakyeewa<br>Mobile platform/ eisumu - whatsup nebindi<br>Online/internet/ kumutimbagano gwainternet<br>Other - specify/ owundi - wandiika………
3.27	In the last 12 months, have you been denied health services?/ Mumyeezi eikumi nebiri egibise, wajaaku mwidwaaliro oba awaidandbibwa awandi walooba kuyambibwa?	single response	Yes/ Yi<br>No/ mbe
3.28	If yes (3,27) Why were you denied health services?/ Nsongaki jebaawa?	single response	I was found too young/ nali muto inho<br>I was told single/ unmarried persons cannot access/ bankoba nti abatali bafumbo tibabawa byakweziyiza kufuna nda<br>I did not have enough money/ tyalinasente dimala<br>Other - specify/ ekindi …………………………….
3.29	If yes (3,05) Have you used condoms in the past 12 months?/ Wakozesaaku akapiira ka galimpitawa oba condom mu myeezi 12 egibise?	single response	Yes/ Yi<br>No/ mbe
3.30	If yes (3,05) I would like to ask you about your recent sexual activity. When was the last time you had sexual intercourse?/ Nendha kubuuza ku bigema ku lwewasembayo okwegaita; li lwewasembayo okwegaita?	integer, single response	Days/ enaku:<br>Weeks/wiki:<br>Months/emyeezi:<br>Years/emyaaka:
3.31	If yes (3,05) The last time you had sexual intercourse with this person, was a condom used?/ Lwewasembayo okwegaita, mwakozesa akapiira kagalimpitawa oba condom?	single response	Yes/ Yi

			No/ mbe	
	Section 4 - Personal attitudes and opinions on contraception: In this section, we wish to follow up with your personal attitudes and opinions regarding SRHR. Mukitundu kino twenda okutegeera ku bwolowoza ku byomukwaano, okwegaita, okuzaala, enkolagana ya abakazi/abawala na basadha/abalenzi, neidembe lyo kubigema kweebyo./			
4.00	Do you agree with the following statements:/ oidha kunkobera oba toikirikiza irara, toikiriza, oliwagati awo (okiriza toikiriza), oikiriza, oba oikirikiza irara. Kwebyo olondaku kirala.		1= strongly disagree/tikirikiza irara | 2= disagree/ tikiriza | 3=neutral/ ndi wagati awo | 4= agree/ ndikiriza | 5= strongly agree/ ndikirikiza irara | DK= don't know/ tidi	
4.01	I find it appropriate for a boy to propose to use a condom./ Nze mbona nti kisaanira omulenzi of kukoba omuwala nti bakozese akapiira oba condom	single response		
4.02	I find it appropriate for a girl to propose to use a condom./ Nze mbona nti kisaanira omuwala okukoba omuwala nti bakozese akapiira oba condom	single response		
4.03	I feel confident that I can use a condom every time if I have sexual intercourse in the future./ Nze nsobola okukozesa akapiira oba condom buli bwenaaba nga negaita nomuntu mumaiso oba yebwida.	single response		
4.04	I am worried about being denied access to SRH services./ Nerarikirira nti bayinza obutampereza oba obutampa bwenendha kumpereza edigema ku byomukwaano, okwegaita, okuzaala, enkolagana ya abakazi/abawala na basadha/abalenzi, neidembe lyo kubigema kweebyo.	single response		
4.05	I am worried about becoming/making someone pregnant early/. Nerarikirira nti ninza okufuna enda nga nkaali kutuusa kiseera/ okunisa omuwala/omukazi enda nga akaali kutuusa kiseera.	single response		
4.06	It is difficult to access contraceptives for me./ Kinzibuwalira okufuna ebyokukozesa okweziyiza okufuna enda.	single response		
4.07	When can a person access a 'modern' form of contraception? (Any form of contraception except natural family planning)/ Mbeeraki oba kiseera ki ekisanira omuntu okufuna oba okukozesa ebigema oba okuziyiza okufuna endha?	single response	Always/ ekiseera kyona kyona nga ayenze	Read the list, tick which one is said
			Never/ wazira naire, talina kufuna	
			Only once married/ nga mufumbo	

			Only after having children/ ngamaze okuzaala abaana	
			At the age of puberty/ nga muvubuka	
			Other - specify/ ekindi …………………………….	
4.08	If a girl becomes pregnant here, whom can she turn to?/ Mukitundu kino, omuwala bwafuna endha, ani gwayinza okutukirira okumuyamba?	multiple response	Parents/ abazaile	Do not read the list, tick which ones are said
			Other family members/ abawaka abandi	
			Health worker/ omusawo	
			Teacher/ abasomesa	
			Traditional leader/ omusawo owekinansi	
			Religious leader/ abakulu abeidini	
			Peer educator/ banange bwetwagaya	
			Partner/boy/girlfriend/ bunange, muganziwe	
			Friends/emikwaano	
			Nobody/ wazira	
			Don't know/ tidhi	
			Other - specify/ owundi - wandiika………	
	Section 5 - Support : In this section, we wish to know about the support that you receive on SRHR issues from those around you. Mukitundu kino, twenda kutegeera okuyambibwa kwofuna okuva kubantu abakwetolweire ku bigema ku bwolowoza ku byomukwaano, okwegaita, okuzaala, enkolagana ya abakazi/abawala na basadha/abalenzi, neidembe lyo kubigema kweebyo			
	How easy do you find it to talk to the following people about sexuality, contraception and relationships? Please rate on a scale of 1-5 how easy it is. 1 being the most difficult and 5 being the easiest./ Kikwanguyira kitya okwogera nabantu bano ku bigema ku byomukwaano, okwegaita, okweziyiza okufuna endha, okuzaala, enkolagana ya abakazi/abawala na basadha/abalenzi, neidembe lyo kubigema kweebyo? Kizibu inho, kizibu, kiri wagati awo, kyangu oba kyangu inho?		Rate<br>1= very difficult/ | 2=difficult| 3=neutral | 4=easy| 5=very easy 1. Kizibu inho 2. kizibu 3.kiri wagati awo 4.kyangu 5. kyangu inho	
5.02	Teachers./ Abasomesa	single response		
5.03	Peers./ Baino, mikwaanogyo byemwaaga	single response		

5.04	Parents. / Abazaire	single response	
5.05	Other family members./ Abantu abandi abawaka nga bagandabo naabolungada	single response	
5.06	Girlfriend/ boyfriend / spouse/ partner./ Muganziwo, omwaami oba omukyaalawo	single response	
5.07	Traditional or community chiefs./ Abakulu abobuwangwa aboomukitundu.	single response	
5.08	(Local) political leader./ Ba LC - abakulu abalonde	single response	
	Do you feel the following people understand you when discussing these topics? Please rate on a scale of 1-5 the level of understanding. 1 being the least understanding and 5 being the most understanding./ Olowooza abantu bano bakutegeera bwooba oyogera ku byomukwaano, okwegaita, okweziyiza okufuna endha, okuzaala, enkolagana ya abakazi/abawala na basadha/abalenzi, neidembe lyo kubigema kweebyo? Tibakutegerera irara, kizibu okukutegeera, kiri wagati awo, bakutegeera muku, bakutegeerera irara?		Rate<br>1= not understanding at all | 2=not so understanding | 3=neutral | 4=understanding | 5=very understanding 1.Tibakutegerera irara, 2. kizibu okukutegeera, 3. kiri wagati awo 4. bakutegeera muku 5. bakutegeerera irara
5.09	Teachers./ Abasomesa	single response	
5.10	Peers./ Baino, mikwaanogyo byemwaaga	single response	
5.11	Parents./ Abazaire	single response	
5.12	Other family members./ Abantu abandi abawaka nga bagandabo naabolungada	single response	
5.13	Girlfriend/ boyfriend / spouse/ partner./ Muganziwo, omwaami oba omukyaalawo	single response	
5.14	Traditional or community chiefs./ Abakulu abobuwangwa aboomukitundu.	single response	
5.15	(Local) political leader./ Ba LC - abakulu abalonde	single response	
	Do you feel supported by the following people in accessing sexuality education and SRH services? Please rate on a scale of 1 to 5. 1 being the least supportive and 5 being the most supportive./ Owulira nti abantu bano bakuyamba oba okuwagira okusomesebwa and kuwerezebwa ku byomukwaano, okwegaita, okweziyiza okufuna endha, okuzaala, enkolagana ya abakazi/abawala na basadha/abalenzi, neidembe lyo kubigema kweebyo?tibakuyamba obakuwagira naire, kizibu okuyamba oba okuwagira, wagati awo, bagezaaku, Bayamba era bawagira inho?		Rate<br>1= very unsupprotive | 2=not so supportive| 3=neutral | 4=supportive| 5=very supportive 1. tibakuyamba obakuwagira naire 2.kizibu okuyamba oba okuwagira 3. wagati awo 4.bagezaaku 5.Bayamba era bawagira inho

5.17	Teachers./ Abasomesa	single response		
5.18	Peers./ Baino, mikwaanogyo byemwaaga	single response		
5.19	Parents./ Abazaire	single response		
5.20	Other family members./ Abantu abandi abawaka nga bagandabo naabolungada	single response		
5.21	Girlfriend/ boyfriend / spouse/ partner./ Muganziwo, omwaami oba omukyaalawo	single response		
5.22	Traditional or community chiefs./ Abakulu abobuwangwa aboomukitundu.	single response		
5.23	(Local) political leader./ Ba LC - abakulu abalonde	single response		
5.25	In general, do you have someone at home with whom you can talk to about your feelings/hopes/worries most of the time?/ Okutwaalira awalala, olinayo omuntu eka gwosobola okwogeraku naye ku bwowulira, byosuubira, ebikwerarikiriza ebiseera ebisinga obungi?	single response	Yes/ Yi	
			No/ mbe	
			Don't know/ tidhi	
5.26	If yes (5,25) Who is that person?/ Naani?	single response	Mother/ maama	Please tick only one option
			Father/ baaba	
			Sister/ mugandawange omuwala	
			Brother/ mugandawange omulenzi	
			Cousin/ mugandawange ku maama,baama omuto oba mwaana wa seenga oba kojja	
			Grandfather/ daada omusadha	
			Grandmother/ daada omukazi	
			Husband/ omwaami	
			Wife/omukyaala	
			Aunt/ ssenga oba maama omuto	
			Uncle/ kojja oba baaba omuto	
			Other - specify/ owundi - wandiika………	
	Section 6 - Violence: In this section, we invite you to share if you have experiences any violence. Mukitundu kino twendha otukobere ku byobaire obitamu kungeri yokubisibwa obubi.			

6.01	How often have you been physically hurt/hit by a member of the opposite sex?/ Mulundi emeka nga okubibwa omunt atali wakikula kyo (omusadha/mulenzi oba omukazi/muwala)? Ebiseera byona byona, ebiseera ebisinga, ebiseera ebindi, kirwaawo okubaawo oba tikibangawo?	single response	All the time/ bulikiseera	Read the list, tick one
			Frequently/ ebiseera ebisinga obungi, emirundi mingi	
			Sometimes/ ebiseera ebindi	
			Rarely/ lulala na lulala	
			Never/ tibankubangaku	
			I don't wish to share this/ tyenda kukyogeraku	
6.02	How often do you experience sexual harassment?/ Mulundi emeka nga abantu bakola ebintu ebiraga nti benda kukukabasanya? Ebiseera byona byona, ebiseera ebisinga, ebiseera ebindi, kirwaawo okubaawo oba tikibangawo?	single response	Everyday/ bulilunaku	Read the list, tick one
			Once or twice a week/ mulundi mulala mu wiki	
			Once or twice a month/ mulundi mulala mumweezi	
			Less than once a month/ tikiweza mulundi mulala mu mweezi	
			Never/ tikintuukangaku	
			I don't wish to share this/ tyenda kukyogeraku	
6.03	Have you ever been physically forced to perform any sexual acts you did not want to?/ Wakakibwaku omukwaano/okwegaita no muntu oba okukola bintu ebyefananiza no kwegaita nga toyenda?	single response	Yes/ Yi	
			No/ mbe	
			No answer/ taizeemu	
6.04	If yes (6,03) Did you turn to someone in case of sexual assault, harassment or force?/ Wa loopa oba wakoberaku omuntu yena yena?	single response	Yes/ Yi	
			No/ mbe	
			No answer/ taizeemu	
6.05	If yes (6,03) Who did you turn to?/ Aani gwe wa loopera oba gwewakobera?	multiple response	Parents/ abazaile	Do not read the list, tick the ones said
			Other family members/ abawaka abandi	

			Religious leader/ abakulu abeidini<br>Health worker/ omusawo<br>Friends/emikwaano<br>Peer educator/ counsellor/ banange bwetwagayaga oba abudhabudha<br>Youth club/ ekibiina kyabavubuka<br>Teacher/ abasomesa<br>Traditional leader/ omusawo owekinansi<br>Police<br>Other - specify/ owundi - wandiika………
	Do you agree with the following statements:/ Nkobera bwolowooza ku bino; toikirikiza irara, toikiriza, oliwagati awo, okiriza oba oikiriza inho?		1= strongly disagree/tikirikiza irara | 2= disagree/ tikiriza | 3=neutral/ ndi wagati awo | 4= agree/ ndikiriza | 5= strongly agree/ ndikirikiza irara | DK= don't know/ tidi
6.06	It is sometimes okay to physically beat or punish a girl if she dishonours her family./ Ebiseera ebindi, kiba kituufu omuwala okukubibwa bwaaba aswaziza abomumaka mwaava	single response	
6.07	It is sometimes okay to physically beat or punish a boy if he dishonours his family. physically beat or punish a girl if she dishonours her family./ Ebiseera ebindi, kiba kituufu omulenzi okukubibwa bwaaba aswaziza abomumaka mwaava	single response	
6.08	If a girl refuses to have sex with her boyfriend, its okay for him to use force or pressure./ Wazira nsonga omulenzi okukoseza amaani oba okukaka muganzi we omukwano omuwala bwaaba alobye	single response	
	If a boy refuses to have sex with his girlfriend, its okay for her to use force or pressure./ Wazira nsonga omuwala okukoseza amaani oba okukaka muganzi we omukwano omulenzi bwaaba alobye	single response	
6.09	A husband is justified in hitting or beating his wife if she argues with him./ Kituufu oba omwaami mutuufu okukuba omukyaalawe omukyaala bwawakana naye?	single response	
6.10	A wife is justified in hitting or beating her husband if he argues with her./ Kituufu oba omukyaala mutuufu okukuba omwaamiwe omwaami bwawakana naye?	single response	

	Section 7 - Gender: In this section, we wish to know about your opinions on gender. Mukitundu kino twendha kutegeera ku byolowooza ku kikula kyabantu, nengeri gyebakolaganamu./ Do you agree with the following statements: Nkobera bwolowooza ku bino; toikirikiza irara, toikiriza, oliwagati awo, okiriza oba oikiriza inho?		1= strongly disagree/tikirikiza irara | 2= disagree/ tikiriza | 3=neutral/ ndi wagati awo | 4= agree/ ndikiriza | 5= strongly agree/ ndikirikiza irara | DK= don't know/ tidi
7.01	Boys/ men are responsible to earn for the household./ Abalenzi oba abasaadha bavunanizibwa okukola okulabirira amaka.	single response	
7.02	Girls/ women have to cook and clean for the household./ Abawala oba abakazi bavunanizibwa okufumba, nokulongoosa waka.	single response	
7.03	The participation of the father is important in raising children./ Kikulu inho baaba okwenigira mu kukuza abaana.	single response	
7.04	A couple should decide together if they want to have children./ Omwaami nomukyaala balina okusalawo bombi oba benda okuzaala abaana.	single response	
7.05	Men should have the final word about decisions in the household./ Abaami oba abasadha bandibaire nokusalawo okwenkomerero mu maka.	single response	
7.06	Boys/ men are not supposed to cry./ Abalenzi oba abasadha tibalina kulira.	single response	
7.07	Girls/ women should always obey men./ Abawala oba abakazi balina okugondara nokukola abasadha byebabakoba.	single response	
7.08	When money is scarce, boys should be send to school before girls./ Esente bwediba tidimala, abaan abalenzin be bandi soose okutwaala kwisomero (abawala balinda).	single response	
	Section 8 - Rights and discrimination: In this section, we wish to know about your opinions on rights and discrimination. Mukitundu kino twendha okutegeera ku byolowooza kwidembe lyabantu nokusosolebwa./		

	Do you agree with the following statements: Nkobera bwolowooza ku bino; toikirikiza irara, toikiriza, oliwagati awo, okiriza oba oikiriza inho?		1= strongly disagree/tikirikiza irara | 2= disagree/ tikiriza | 3=neutral/ ndi wagati awo | 4= agree/ ndikiriza | 5= strongly agree/ ndikirikiza irara | DK= don't know/ tidi
8.01	Contraceptives should be available for unmarried young people./ Abantu abatali bafumbo bandikirizibwa okufuna ebikozesebwa okugema oba okuziyiza okufuna endha.	single response	
8.02	A schoolboy who makes a schoolgirl pregant should be expelled from school./ Omulenzi omusomi bwafunisa omuwala omusomi endha agobebwe mwissomero	single response	
8.03	A schoolgirl who gets pregnant should be expelled from school. Omuwala asoma bwafuna enda agobebwe mwisomero.	single response	
8.04	I should have the choice to decide whom to marry./ Ndina eidhembe okusalawo gwenenda okufumbirwa.	single response	
8.05	I would be ashamed if someone in my family had HIV./ Kyandinswaziza omu kuba family oba ewaife okuba nga yalwaala silimu.	single response	
8.06	I find it okay that children living with HIV go to school with children who are HIV negative./ Kunze, wazira nsonga/mutawaana abaana abalwaire ba siliimu okuba mwisomero eirara nabazira siliimu.	single response	
	Scrtion 9 - Sexuality: In this section, we wish to know about your opinions on sexuality./ Mukitundu kino twendha okutegeera ku byolowooza byomukwaano, okwegaita, okuzaala, enkolagana ya abakazi/abawala na basadha/abalenzi.		
	Do you agree with the following statements:/ Nkobera bwolowooza ku bino; toikirikiza irara, toikiriza, oliwagati awo, okiriza oba oikiriza inho?	single response	1= strongly disagree/tikirikiza irara | 2= disagree/ tikiriza | 3=neutral/ ndi wagati awo | 4= agree/ ndikiriza | 5= strongly agree/ ndikirikiza irara | DK= don't know/ tidi
9.01	I feel/ felt afraid about the changes that occur in my body due to puberty./ Mpulira/nawulira okutya omubili gwange bwegukyuka/bwegwakyuka mu buvubuka.	single response	
9.02	I feel guilty / ashamed when I have sexual feelings./ Mpulira okuswaala oti nina omusango bwempulira okwaaka.	single response	

9.03	I feel I am able to make the decision myself if I want to have sex or not./ Mpulira nga nsobola okwesalirawo oba negaita nomuntu oba mbe.	single response	
9.04	(Only for married resondents) Men and women should know what his or her partner likes during sex./ Abaami nabakyaala bandyetaaze okutegeera banaibwe mumukwaano byebetaaga nga begaita	single response	
9.05	(Only for married resondents) It is important to have pleasure during sex./ Kikulu okunumirwa omukwaano mukwegaita	single response	
9.06	I feel confident about how my body looks./ Mpulira bulungi, tiswaala kungeri omubirigwange bwegufanana.	single response	
9.07	I am able to express my feelings about sexuality and relationships./ Nsobola okwogera kubwempurira ku byomukwaano, okwegaita, okuzaala, enkolagana ya abakazi/abawala na basadha/abalenzi, neidembe lyo kubigema kweebyo.	single response	
	Section 10 - General empowerment: In this section, we wish to know about your thoughts on empowerment. Mukitundu kino twenda okutegeera byolowooza ku maani, obuyinza, nobuvumu okusobola okweyamba not kuyamba abantu abandi.		
10.01	Who decides how many children you will have (in the future) ?/ Ani asalawo oba alisalawo kumuwendho gwaabaana bozaala/bolizaala.	single response	Me/ ninze
			My husband/ wife/ omwaami wange oba omukyaala
			My in-laws/ abazaala, manina oba baganda boomwaami oba omukyaala
			My family/ abewange gyenzaalibwa
			Me and my partner/ nze no mwaami, omukyaala oba muganzi wange
			Other - specify/ owundi - wandiika………
10.02	Are you member of a youth club?/ Alinayo ekibiina kyabavubuka kyolimu nga memba?	single response	Yes/ Yi
			No/ mbe
10.03	If yes (10,02) How do you benefit from being a member of this club?/ Oganwirwamu ki mukibiina kino?	multiple response	Making friends/ kukola mikwaano

			Discussing (sensitive) issues/ okwogera kubintu ebyekyaama oba ensoni ebitatera kwogerwaaku<br>Advice/ okufuna amagezi
			Sharing skills/ okukabana nokweega ebyokukola
			Other - specify/ ekindi ....
10.04	(If in school) Do you feel that you can influence the decisions made at schools/ in class?/ Owulira nti osobola okuwulirizibwa mukusalawo ebigema kwisomero oba mukibiina kyo?	single response	Yes/ Yi
			No/ mbe
			Don't know/ tidhi
10.05	Do you feel that you can influence the decisions made in your household?/ Owulira nti osobola okuwulirizibwa mukusalawo ebigema waka?	single response	Yes/ Yi
			No/ mbe
			Don't know/ tidhi
10.06	Do you feel that you can influence the decisions made in your community?/ Owulira nti osobola okuwulirizibwa mukusalawo ebigema kukitundu kyo?	single response	Yes/ Yi
			No/ mbe
			Don't know/ tidhi
10.07	Who usually makes decisions about your health care ?/ Aani asalawo kubigema kubyo kuidandabibwako?	single response	Myself/ nze mweene
			Me and someone else jointly/ nze nomuntu owundi
			Someone else/ omuntu owundi
10.08	If someone else involved (10,07) Who is that person?/ Aani asalawo?	single response	Husband/ wife/ omwaami, omukyaala
			Other family member/ omuntu owundi owawaka<br>Other - specify/ owundi - wandiika.........
	Do you agree with the following statements:/ Nkobera bwolowooza ku bino; toikirikiza irara, toikiriza, oliwagati awo, okiriza oba oikiriza inho?		1= strongly disagree/tikirikiza irara | 2= disagree/ tikiriza | 3=neutral/ ndi wagati awo | 4= agree/ ndikiriza | 5= strongly agree/ ndikirikiza irara | DK= don't know/ tidi
10.09	I feel that my opinions have value./ Mpulira nti byendowooza ni byendogera bilina omugaso.	single response	

10.10	I decide for myself who to date./ Nesalirawo gwenenda okwenda.	single response	
10.11	If I see something wrong in school or the neighborhood I feel confident to take action to help./ Bwewabaawo ekitatereire mwisomero oba mu kitundu mwemba, Nina obuvumu okubaaku ni kyenkola okuyamba.	single response	
11	Section 11: Additional questions		
	THANK YOU FOR YOUR TIME. Thank you for staying with us through the questions./ Weebale inho okutuwa ekiseera okwiramu ebibuuzo.		
11.01	Do you have any questions?/ Olina ebibuuzo ?	single response	Yes/ Yi
			no/ mbe
	Comment	Text	
11.02	Interviewer's comments	Text	
