# Supplementary material for: Young people’s choice and voice concerning sex and relationships: effects of the multicomponent Get Up Speak Out! Programme in Iganga, Uganda
Source: BMC Public Health. 2022 Aug 23;22:1603. doi: 10.1186/s12889-022-13919-x (PMC9396562; doi:10.1186/s12889-022-13919-x)
Supplement: Supplementary file 2 — Additional file 2. Topic guide for focus group discussions (FGDs) with young girls and boys (15-24 years old) with Lusoga translations. [file 12889_2022_13919_MOESM2_ESM.pdf]

Additional File 2

Topic guide for focus group discussions (FGDs) with young girls and boys (15-24 years old) with Lusoga translations

Area of inquiry	Guiding Questions	Prompts
Introduction<br>Enandurwa	• Age/emwaake<br>• Sex/ ekikula<br>• Education/obwegerese<br>• Marital status/enemererayo mubwobufumbo<br>• Children/ olina abaana<br>• Since when living here?/wantandiika li okuba mukitundu kino?<br>• With whom are you living?/ oba naani?<br>• Daily business/ okola mulimo ki?<br>• Religion/oliwaidini ki?	
Social norms & attitudes around boys and girls<br>Enono dobuwangwa nendowooza kubawala nabalenzi	1. What is a good girl/boy, characteristics<br>Omuwala olungi mukitundu kino naali atya? Ate omulenzi?<br>2. What kind of desires do young people in this community have in relation to marriage, children & parenting roles?<br>Abavubuka bomukitundu kino, biki byebenda era byebasubira kubyobufumbo, abaana na bazaile okulabirira abaana?	Please give examples<br>Muninonole nga muwa nebyokuboneraku
Empowerment of young people<br>Abavubuka okuba nobuyinza, namaani kubimugemaku	3. What are important subjects for young people to make decisions about?<br>Biki ebikulu abavubuka byebalina okuba nti besalirawo?<br>4. How can young people express themselves on sensitive issues? Abavuka basobola batya okweyogerera oba okulaga kyebalowooza nikyebenda ku bintu ebitayogerwayogerwaku oba ebirowozebwa nti byansoni?<br>5. What kind of space is provided by others (parents/ teachers/health providers/ peers)? Abazaire, abasomesa, abasawo ni mikwaanogyaimwe bwemagayaga babawa akawaganya okwogera nokulaga bwowulira?	Probe on forms or examples of self-expression:<br><br>If others listen to young people<br>Abantu bawuliriza abavubuka?<br>If young people have freedom to move around<br>Abavubuka baline eidembe okutambula mukitundu?<br>If young people can make decisions<br>Abavubuka basobola okwesalirawo kubigema kubulamu bwaibwe?<br>If young people can discuss sexual health<br>Abavubuka basobola okwogera kubigema kubyobulamu kubyokwegaita nokuzaala?<br>If young people can say no to sex<br>Oba abavubuka basobola okuloba okwegaita?<br><br>Are there any changes in the space provided by adults, over the past 3 years?<br><br>Ghaligo edhawulo ku'kaghaganhia akawebwa abantu abakulu, mu'myaka esatu ejibise
Sexual health and practices<br>kubyobulamu kubyokwegaita nokuzaala nebikolebwa	6. When do adolescents start to have sexual relations here?<br>Abavubuka abato (13+) batandiikali okuba nibebenda?<br>7. What are the issues that adolescents face with regard to SRH?	Probe on

Area of inquiry	Guiding Questions	Prompts
	Bizibu abavubuka byebasanga kubigema ki byobulamu nebigama ku ku byomukwaano, okwegaita, okuzaala, enkolagana ya abakazi/abawala na basadha/abalenzi, neidembe kubigema kweebyo?	positive and negative aspects of sexual relations<br>Buuza ku birungi nikubibi ku nkolagana wagati wabawala nabalenzi kubyomukwaano nokegaita.<br><br>Positive and negative issues faced by girls and boys:<br>Ebirungi nikubibi ku nkolagana wagati wabawala nabalenzi ku:<br>Being in love/ okuba mumukwaano<br>Romance/ omukwaano<br>Child marriage/ okufumbiza abaana abakaali kwetuuka<br>Teenage pregnancies/ okufuna enda ngokaalli kuweza mwaaka aabiri<br>STIs/ obulwaire obobukaba<br>Sexual Violence / okukakibwa omukwaano<br>Power relations /okulaga amaani munkolagana<br><br>Did you observe any changes in the issues that adolescents face with regad to SRH in the past 3 years? Why?<br><br>Gha'bonaghoku endhawulo mu'nsonga abavubuka debagana nga digemagan ku SRH, mu'myaka 3 ejibise? Lwaki?
Sources of information on sexual health<br>Webatoola amawulire oba ebitegezebwa ku byobulamu nebigama ku ku byomukwaano, okwegaita, okuzaala.	8. Where do adolescents get SRH information and education?<br>Abavubuka batoolawa ebyokusoma nebyogera/amawulire ku byobulamu nebigama ku byomukwaano, okwegaita, okuzaala, enkolagana ya abakazi/abawala na basadha/abalenzi, neidembe kubigema kweebyo?<br>9. Who play a major role in providing this information?<br>Nibaani abasinga kukola kubyokuwa abavubuka amawulire/ebitegeeza kweebyo?	Please probe on:<br><br>What kind of information they receive and the quality of the information<br>Bwakutegezebwaki/mawulireki namutindoki ogwamawulire/ogwebyokutegezebwa gwebafuna<br><br>Observed changes in SRH information over the past 3 years<br><br>Ghabonagho endhawulo mu'baka bwa SRH, mu'myaka 3 ejibise?<br><br>To whom young people turn to when they have questions on sexual health matters<br>Abavubuka batuukirira ani bwebaba nga balina ebibuuzo ku byobulamu ebigema ku kwegaita no kuzaala?<br><br>And which persons are most influential (health providers,

Area of inquiry	Guiding Questions	Prompts
		teachers, peers, peer educators, parents and why)<br>Bantuki abasinga okusika abantu oba abavubuka ebewuliriramu?<br><br>Observed changes in source of SRH information in the past 3 years<br><br>Ghabonagho endhawulo mu'nsibuko yo bubaka bwa SRH mu'myaka 3 ejibise?
Role of parents and caregivers/ family members<br>Abazaile, abalabirira abaana, nabawaaka bwebavunaanizibwaki	10. What is the role of parents and caregivers in relation the sexual health of young people?<br>Abazaile, abalabirira abaana, nabawaaka bwebavunaanizibwaki kubigema ku byomukwaano, okwegaita, okuzaala kwabavubuka?	Probe for examples
Sexual violence<br>Okukakibwa omukwaano	11. Is sexual violence taking place?<br>Okukakibwa omukwaano kuliwo mukitundu kino?<br>12. What kind of sexual violence and who is mainly affected?<br>Kukakibwa omukwaano kwangeriki? Ani asinga okukosebwa? Ani gwebasinga okukaka omukwaano?<br>13. What kind of support is provided to prevent and address sexual violence?<br>Kiki ekikolebwa okubona nti okukakibwa omukwaano tikubaawo?	Probe on kind of perpetrators, vulnerable groups of young people facing sexual violence<br>Sanctions by society and laws regulation?<br>Access to counselling services<br>Buuza ki bika bya abakaka abavubuka omukwaano, abasinga okukabasanizibwa; emateeka nebibonerezo ebiriwo; oba waliwo abababudhabudha.<br><br>Observed changes in sexual violence in past 3 years<br><br>Ghabonagho endhawulo mubyokukaka omukwano mu'myaka 3 ejibise
HIV<br>Siliimu	14. What kind of services and information is required for girls and boys to prevent HIV infection?<br>Mperezaki, nobyokutegeera oba mawulire ki obwetagisa okusoboza abawala nabalenzi okweziyiza okufuna siliimu?<br>15. What kind of services are available for young people living with HIV?<br>Mperezaaki eriwo mukitundu kino okuyamba abavubuka abalina obulwaire bwa siliimu?	Probe on:<br>Different needs and perspectives of girls, boys vulnerable to HIV infection<br>What specific services are required for young people that live with HIV? (Use of viral load testing)<br>Buuza ku ebyetaago nendowooza ya bawala nabalenzi abayinza okugemebwa siliimu amangu.<br>Mperezaaki edeetagibwa abavubuka abalina akawuuka ka siliimu (nga okukebera obutaafali)<br><br>Possible changes observed in availability and quality of HIV services over the past 3 years<br><br>Ghabonagho endhawulo mu kubagho nhi mu mutindo gwe'mpereza dha HIV mu'myaka 3 ejibise?

Area of inquiry	Guiding Questions	Prompts
Sexual Health Services<br>Empereza kubyomukwaano, okwegaita, nokuzaala.	16. How do young people get access to SRH services?<br>Abavubuka bafuna batya empereza oba obwidandabi obugema ku byobulamu nebigama ku ku byomukwaano, okwegaita, okuzaala, enkolagana ya abakazi/abawala na basadha/abalenzi, neidembe kubigema kweebyo ebya bavubuka?<br>17. What kind of SRH services can young people access?<br>Mperezaki kubyobulamu nebigama ku byomukwaano, okwegaita, okuzaala, enkolagana ya abakazi/abawala na basadha/abalenzi, neidembe kubigema kweebyo ebya bavubuka byebayinza okufuna mukitundu kino?	Probe on accessibility for young people in relation to identity (age, gender, class, urban/rural, HIV status)<br>And referral systems/ quality of services<br>Buuza oba empeereza ekyuukamu mubavubuka okusinziira ku myaaka, ekikula, ebyenguna, oba bamukyaalo oba mutown, oba balina siliimu oba bbe.<br>Oba balagirirwa awandi bwebaba betaaga empeereza esingawo; omutindo gwe empeereza<br><br>Probe on contraceptives and attitude of service providers<br>Buuza ku bikozesebwa okweziyiza okufunal enda; endowooza ya basawo kukino.<br><br>Changes observed in access to SRH services for youth over the past 3 years<br><br>Endhawulo edabonebwa mu'kutukirila empereza dha SRH mu'bavubuka mu'myaka 3 ejibise?
Role of peer educators<br>Obuvunanizibwa bwabavubuka ebeegereza abandi.	18.What is the role of peer educators in supporting the sexual health of boys and girls?<br>Bavubuka banaimwe ababasomesa, bikibyebakola mukuyamba abavubuka abawala nabalenzi ku byomukwaano, okwegaita, nokuzaala.	Probe on both positive and negative contributions<br>Attitude towards young people<br>Referral<br>Knowledge<br>Buuza kubirungi nebibi byebakola; endowooza yaibwe nengeri gyebabisaamu abavubuka; oba balagirira abavubuka wawookutoola byebetaaga; oba baidhi byebakola.<br><br>Any changes over the past 3 years?<br>Endhawulo edabonebwa mu'myaka 3 ejibise?
Role of health workers<br>Obuvunanizibwa bwababawasawo	19. Where are the health facilities here?<br>Which types?<br>Amadwaaliro galiwa mukitundu kino? Gabikaaki?<br><br>20. What is the role of health workers in providing youth friendly SRH services<br>Abasawo balina buvunanizibwaki kukubona nti abavumuba bafuna empereza ku byomukwaano, okwegaita, okuzaala, enkolagana ya abakazi/abawala na basadha/abalenzi, neidembe kubigema kweebyo mungeri abavubuka gyebenda, etabasosola?	What is the role of health workers (in positive and negative contributions)<br>Who does the referral between the community and health facilities?<br>Abasawo balina buvunanizibwaki? birungi nebibi byebakola? Nibaani mubasawo alagirira abavubuka mukitundu amalwariro agokujaamu?<br><br>Observed changes in the role of health workers over the past 3 years<br><br>Endhawulo edabonebwa mu'mirimo gya abasawo mu'myaka 3 ejibise?
Role of teachers and schools	21. What is the accessibility and quality of schools?	Safety at schools. Policies on sex education

Area of inquiry	Guiding Questions	Prompts
Obuvunanizibwa bwabasomesa namasomero	Mulina amasomero ameka mukitundu kino? Galigatya kubyomutindo? Abavubuka kibanguyira okuja nokuba mumasomero?<br>22. What is the role of teachers in SRHR and sexuality education?<br>Abasomesa balina buvunanizibwaki okubona nti abaana bafuna okwegeresebwa ku byomukwaano, okwegaita, okuzaala, enkolagana ya abakazi/abawala na basadha/abalenzi, neidembe kubigema kweebyo?	Social and cultural acceptability of girls and boys receiving sexuality education<br>Role of teachers in discussing sensitive issues<br>Obukuumi kumasomero. Amateeka agagema kubyokwegeresa kubyomukwaano nokwegaita. Oba abawala nabalenzi baikirizibwa okusomesebwa kubyomukwaano nokwegaita.<br>Abasomeza balina buvunanizibwaki oba, bakolaki kukwogera kubintu ebitatera kwogerwaku, ebyekyaama.<br><br>Any changes in the role of teachers in SRHR over the past 3 years?<br>Endhawulo edabonebwa mu'mirimo gya basomesa kubya SRHR mu'myaka 3 ejibise?
Mechanisms	23. What would help to give girls and boys a better future?<br>Kiki ekiyinza okukolebwa okuyamba abawala nabalenzi okufuna obulamu obutangaavu mumaiso?	Probe for examples<br>Buuza ebyokuboneraku
Questions	24. Anything else you like to share? Questions for us?<br>Eriyo ekindi kyoyenda okutukobera? Olina ebibuuzo?	

Topic guide for semi-structured interviews (SSIs) with girls and boys (15-24 years old) (similar to above)

Topic guide for semi-structured interviews (SSIs) with parents and caregivers, religious and traditional leaders, teachers, health workers and peer educators (similar to above)

Topic guide for key informant interviews (KIIs) with NGO staff, youth networks, health care providers and policy makers at the county level (similar to above; additions indicated below)

Role of NGO's	What can NGO's do to improve and support the sexual and reproductive health of girls and boys?<br>Biki ebibiina ebyanakyeewa byebiyinza okukola okulongosa oba okutumbula mu bigema ku byomukwaano, okwegaita, okuzaala, enkolagana ya abakazi/abawala na basadha/abalenzi, neidembe kubigema kweebyo ebyabawala nabalenzi?	Probe on both positive and negative contributions<br>Attitude towards young people<br>Referral<br>Knowledge<br>other<br>Buuza kubirungi nebibi byebakola; endowooza yaibwe nengeri gyebabisaamu abavubuka; oba balagirira abavubuka wawookutoola byebetaaga; oba baidhi byebakola.<br><br>Probe on the role of NGOs involved in GUSO<br><br>Buuza ku'migaso gye bitongole ebyenigira mu GUSO
Role of Policy Makers	What kind of legislation is in place to support the sexual and reproductive health of girls and boys?<br>Mateekaki getulina agayamba ku byomukwaano, okwegaita, okuzaala, enkolagana ya abakazi/abawala na	Probe on legislation and implementation<br>Buuza ku mateeka nengeri gyegateebwa munkola

	basadha/abalenzi, neidembe kubigema kweebyo ebyabawala nabalenzi?<br><br>What other actions are taken or can be done to create a conducive environment for girls and boys to realise their SRH?<br>Biki ebindi ebikolebwa oba ebiyinza okukolebwa okutaawo embeera eyamba abawala nabalenzi ku byomukwaano, okwegaita, okuzaala, enkolagana ya abakazi/abawala na basadha/abalenzi, neidembe kubigema kweebyo?	Over the past 3 years, have there been any changes in legislation or policies? Which ones and why?<br><br>Mu’myaka 3 ejibise, ghabeireghoku endhawulo mu mateka? Mu’galigha ate lwaki
