# Supplementary material for: Young people’s choice and voice concerning sex and relationships: effects of the multicomponent Get Up Speak Out! Programme in Iganga, Uganda
Source: BMC Public Health. 2022 Aug 23;22:1603. doi: 10.1186/s12889-022-13919-x (PMC9396562; doi:10.1186/s12889-022-13919-x)
Supplement: Supplementary file 3 — Additional file 3. Description of outcome variables. [file 12889_2022_13919_MOESM3_ESM.pdf]

Additional file 3

Description of outcome variables

Variable	Definition	Question asked in the survey	Response options
Sex and relationships			
Ever had sexual intercourse	Percentage of youth (15-24 years) who have ever had sexual intercourse	Have you ever engaged in sexual intercourse	Yes (1)<br>No (0)
Self-expression			
I am able to express my feelings about sexuality and relationships	Percentage of youth (15-24 years) who agreed (strongly agreed or agreed that they were able to express their feelings about sexuality and relationships	Do you agree with the following statement: I am able to express my feelings about sexuality and relationships	Strongly disagree (1)<br>Disagree (2)<br>Neutral (3)<br>Agree (4)<br>Strongly agree (5)<br>Not applicable (NA)
Ease in talking to these stakeholders about sexuality, contraception and relationships	Percentage of youth (15-24 years) who felt at ease (easy and very easy) to talk to stakeholders about sexuality, contraception and relationships	How easy do you find it to talk to the following people about sexuality, contraception and relationships?<br>Please rate on a scale of 1-5 how easy it is. 1 being the most difficult and 5 being the easiest.<br>Health workers<br>Teachers<br>Girlfriend/boyfriend/spouse/partner<br>Peers<br>Parents<br>Other family members<br>Religious leaders<br>Traditional or community chiefs<br>(Local) political leader<br>Traditional or community chiefs<br>(Local) political leader	Very difficult (1)<br>Difficult (2)<br>Neutral (3)<br>Easy (4)<br>Very easy (5)<br>Not applicable (NA)
Decision-making			
I decide for myself who to date	Percentage of youth (15-24 years) who agree (strongly agree or agree) that they can decide for themselves whom to date	Do you agree with the following statement: I decide for myself who to date	Strongly disagree (1)<br>Disagree (2)<br>Neutral (3)<br>Agree (4)<br>Strongly agree (5)<br>Not applicable (NA)
I should have the choice to decide whom to marry	Percentage of youth (15-24 years) who agreed (strongly agreed or agreed) that they should have the choice whom to marry	Do you agree with the following statement: I should have the choice to decide whom to marry	Strongly disagree (1)<br>Disagree (2)<br>Neutral (3)<br>Agree (4)<br>Strongly agree (5)<br>Not applicable (NA)
I feel confident that I can use a condom every time I have sexual intercourse in the future	Percentage of youth (15-24 years) who agreed (strongly agreed or agreed) that they feel confident that they can use a condom every time they have sexual intercourse	Do you agree with the following statement: I feel confident that I can use a condom every time I have sexual intercourse	Strongly disagree (1)<br>Disagree (2)<br>Neutral (3)<br>Agree (4)<br>Strongly agree (5)<br>Not applicable (NA)

I find it appropriate for a boy to propose to use a condom	Percentage of youth (15-24 years) who agreed (strongly agreed or agreed) that they find it appropriate for a boy to propose condom use	Do you agree with the following statement: II find it appropriate for a boy to propose condom use	Strongly disagree (1)<br>Disagree (2)<br>Neutral (3)<br>Agree (4)<br>Strongly agree (5)<br>Not applicable (NA)
I find it appropriate for a girl to propose to use a condom	Percentage of youth (15-24 years) who agreed (strongly agreed or agreed) that they find it appropriate for a girl to propose condom use	Do you agree with the following statement: II find it appropriate for a girl to propose condom use	Strongly disagree (1)<br>Disagree (2)<br>Neutral (3)<br>Agree (4)<br>Strongly agree (5)<br>Not applicable (NA)
A couple should decide together if they want to have children	Percentage of youth (15-24 years) who agreed (strongly agreed or agreed) that a couple should decide together if they want to have children	Do you agree with the following statement: I decide for myself who to date	Strongly disagree (1)<br>Disagree (2)<br>Neutral (3)<br>Agree (4)<br>Strongly agree (5)<br>Not applicable (NA)
Men should have the final word about decisions in the household	Percentage of youth (15-24 years) who agreed (strongly agreed or agreed) that men should have the final word about decisions in the household	Do you agree with the following statement: Men should have the final word about decisions in the household	Strongly disagree (1)<br>Disagree (2)<br>Neutral (3)<br>Agree (4)<br>Strongly agree (5)<br>Not applicable (NA)
I worry about being denied access to contraceptives	Percentage of youth (15-24 years) who agreed (strongly agreed or agreed) that they worry about being denied access to contraceptives	Do you agree with the following statement: I worry about being denied access to contraceptives	Strongly disagree (1)<br>Disagree (2)<br>Neutral (3)<br>Agree (4)<br>Strongly agree (5)<br>Not applicable (NA)
Currently use contraception	Percentage of youth (15-24 years) who (currently) use contraception at the time of the survey	Do you currently use any contraception/family planning methods?	Yes (1)<br>No (0)
Ever used SRH services	Percentage of youth (15-24 years) who ever used SRH services	In general, which of the following services have you used? (can be before COVID-19)	Yes (1)<br>No (0)
I worry about being denied access to SRH services	Percentage of youth (15-24 years) who agreed (strongly agreed or agreed) that they worry about being denied access to SRH services.	Do you agree with the following statement: I worry about being denied access to SRH services	Strongly disagree (1)<br>Disagree (2)<br>Neutral (3)<br>Agree (4)<br>Strongly agree (5)<br>Not applicable (NA)
Support felt by the following people in accessing sexuality education and SRH services	Percentage of youth who felt supported (supported and very supported) by stakeholders in accessing sexuality education and SRH services.	Do you feel supported by the following people in accessing sexuality education and SRH services? Please rate on a scale of 1 to 5. 1 being the least supportive and 5 being the most supportive.<br>Health workers<br>Teachers<br>Girlfriend/boyfriend/spouse/partner<br>Peers<br>Parents<br>Other family members<br>Religious leaders<br>Traditional or community chiefs<br>(Local) political leader	Not supportive at all (1)<br>Not so supportive (2)<br>Neutral (3)<br>Supportive (4)<br>Very supportive (5)<br>Not applicable (NA)

		Traditional or community chiefs<br>(Local) political leader	
