# Supplementary material for: Young people’s choice and voice concerning sex and relationships: effects of the multicomponent Get Up Speak Out! Programme in Iganga, Uganda
Source: BMC Public Health. 2022 Aug 23;22:1603. doi: 10.1186/s12889-022-13919-x (PMC9396562; doi:10.1186/s12889-022-13919-x)
Supplement: Supplementary file 4 — Additional file 4. The Regression Model. [file 12889_2022_13919_MOESM4_ESM.pdf]

Additional file 4

The Regression Model

The regression model used was:

$$\text{logit}(P(\gamma=1)= \beta0+ \beta1*[\text{time}]+ \beta2* [\text{GUSO}] + \beta3*[\text{GUSO}*\text{time}]+ \beta4*[\text{covariate1}]\ldots+\beta x*[\text{covariateX}]+ \varepsilon$$

In the model, У represents the binary outcome; β0 is the baseline odds of outcome variable at baseline in the control area, β1 quantifies the trend over time in the intervention area. I.e. the odds in the intervention area over time. In the tables it is referred to as 'Time'; β2 quantifies the difference between the intervention and control area at baseline. I.e. the odds in the control area at baseline vs the odds in the intervention area at baseline. In the tables it is referred to as 'GUSO intervention'. β3 quantifies the difference between the expected trend in the intervention area if it would follow the same trend as the control area and the observed trend. In the tables it is referred to as 'GUSO : Time'. If this variable is significant, it means that the trend in the intervention area is statistically significantly different from the trend in the control area. Lastly, ε represents the random error term.
